# Recent Advances in Strategies for Improving the Performance of CO_2_ Reduction Reaction on Single Atom Catalysts

**DOI:** 10.1002/smsc.202000028

**Published:** 2020-12-16

**Authors:** Qiyou Wang, Chao Cai, Minyang Dai, Junwei Fu, Xiaodong Zhang, Huangjingwei Li, Hang Zhang, Kejun Chen, Yiyang Lin, Hongmei Li, Junhua Hu, Masahiro Miyauchi, Min Liu

**Affiliations:** ^1^ Shenzhen Research Institute School of Physics and Electronics Central South University Changsha 410083 Hunan P. R. China; ^2^ College of Materials Science and Engineering Hunan Province Key Laboratory for Advanced Carbon Materials and Applied Technology Hunan University Changsha 410082 Hunan P. R. China; ^3^ School of Materials Science and Engineering Zhengzhou University Zhengzhou 450001 Hunan P. R. China; ^4^ Department of Materials Science and Engineering School of Materials and Chemical Technology Tokyo Institute of Technology Tokyo 152‐8503 Japan

**Keywords:** central atoms, coordination environment, CO_2_ reduction, electrocatalysts, single atom catalysts

## Abstract

Excessive consumption of fossil fuels gives rise to the increasing emission of carbon dioxide (CO_2_) in the atmosphere and furthers the ecocrisis. Electrochemical CO_2_ reduction (ECR) has both functions of dwindling greenhouse gas concentration and converting it into valuable products. Due to the intrinsic chemical inertness of CO_2_ molecules, the study on efficient and low‐cost catalysts has attracted much attention. Recently isolated atoms, dispersed in stable support, play an important role in decreasing energy barriers of intermediate steps and obtaining target products with high activity and selectivity for ECR. The effective regulation of central atoms or coordination environment is significant to realize the desired performances of ECR with a high efficiency and selectivity. Hence, a comprehensive summary about strategies for improving the performance of ECR on single atom catalysts (SACs) is necessary. Herein, the SACs on various supports are introduced, the methods to design stable SACs are discussed, and the strategies for tuning the performance of ECR on SACs are summarized. The localized environment manipulation is widely used for high‐performance SACs design, including regulating central atoms and coordination environment. Finally, the perspectives are discussed to shed light on the rational design of intriguing SACs for ECR.

## Graphical abstract

The performance of single atom catalysts (SACs) in carbon dioxide (CO_2_) electroreduction is closely related to the central atoms and its coordination environment. Strategies which include regulating the central atoms, coordination atoms, coordination number, and diatomic strategies are successfully developed to enhance the performance of SACs in CO_2_ reduction reaction.

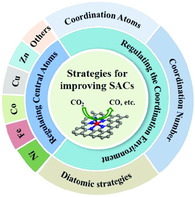

## 1 Introduction

**Figure 1:**
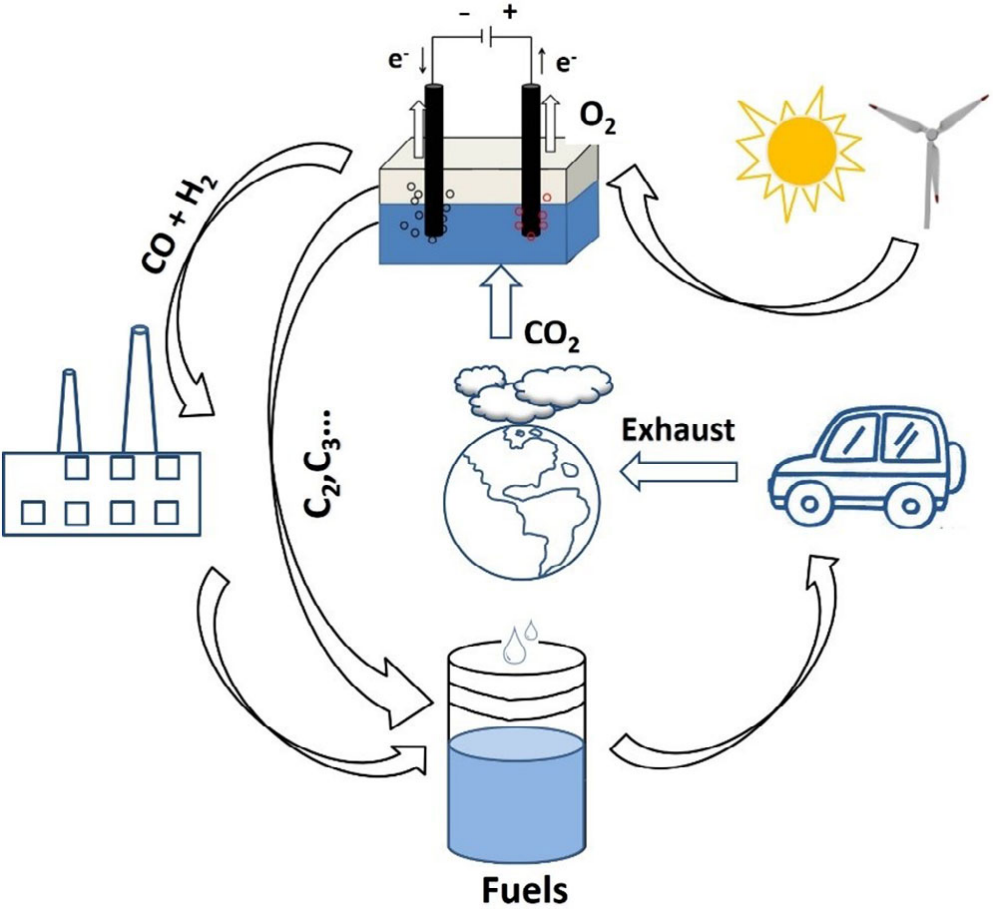
The scheme shows a sustainable carbon recycling system and carbon neutral process.

The concentration of CO_2_ in atmosphere and ocean has increased from approximately 280 ppm in the early 1800 s to 385 ppm now.^[^ ^1^ ^]^ Negative effects of climate change including global warming^[^ ^2^ ^]^ and ocean acidification^[^ ^3^ ^]^ are brought by increase in CO_2_ emission from consuming fossil fuels.^[^ ^4^ ^]^ Nonetheless, now there is no eligible and reliable substitute for fossil fuels, meaning that it will likely continue to be a major source of energy in the forseeable future[^2^, ^5^] If nothing is done, the concerntration of CO_2_ will continue to rise to 600 ppm in the end of the 21st century.^[^ ^6^ ^]^ To solve the aforementioned problems, numerous studies have focused on conversion of CO_2_ into other value‐added products, such as formate, alcohols, and hydrocarbon (HC).[^2^, ^6^, ^7^] Establishing such an artificial sustainable carbon recycling system and carbon neutral process possibly offer reliable solutions to reduce the concentration of CO_2_ in atmosphere and ocean (**Figure** **1**).

In the sustainable “CO_2_ economy”, CO_2_ can be converted into valuable products mainly through four approaches, including electrochemical, biological, biochemical, photochemical, and thermochemical reactions.[^3^, ^4^, ^8^] Among these reactions, direct electroreduction of CO_2_ (ECR) into formate, alcohols, and HC aroused widespread interest.^[^ ^9^ ^]^ It is considered as one of the most promising carbon capture and utilization (CCU) strategies because of three aspects.^[^ ^10^ ^]^ 1) The condition that ECR can be operated under ambient temperature and pressure is exceeding mild, which facilitates the establishment of modular and scalable equipment for industry.^[7b]^ 2) Diverse selectivity of ECR can be obtained by adjusting intrinsic characteristics of catalysts and external parameters of ECR cell.[^5^, ^11^] 3) Environmental and economic feasibility enhance the value of ECR.^[6a]^ Products from ECR such as ethylene or syngas could be involved in carbon recycling system as carbon sources again.^[^ ^11^ ^]^ The pathway of ECR to chemicals with added value simultaneously alleviate the excessive CO_2_ emission and offer an opportunity for renewable energy storage.^[2a]^

Recently, single‐atom catalysts (SACs) featuring isolated or coupled metal atoms uniformly dispersed on a conductive support have recently become eye‐catching in ECR.[^1^, ^12^] Comparing to the bulk/nano‐catalysts used in ECR, no metal–metal bond shows in SACs, ensuring the highly charged isolated atoms achored on/in supports.^[^ ^13^ ^]^ In addition, the SACs have advanced advantages in accelerating charge transfer, feasible regulating active sites and maximizing atom utilization, which integrate the superiority of both homogeneous and heterogeneous catalysts. Varela et al. demonstrated the similarity between the heterogeneous charge‐transfer reaction mechanism and that of molecular metal−nitrogen porphyrin‐type macrocyclic complexes strongly, suggesting that the Fe‐N‐C embeded in the solid‐state electrocatalyst act as the primary active center of ECR.[^2^, ^14^] In 1970s, ECR was conducted first using well‐defined M‐N_4_ sites. Since then, numerous catalysts contain M‐N_4_ have been studied in either homogeneous catalysts or heterogeneous reactions.^[^ ^15^ ^]^ More than a dozen kinds of SACs, with high ECR performance, have been screened out by experiments and theoretical studies. CO_2_ on SACs is converted into C_1_ products principally (such as formate, CO, and CH_4_), because the neighboring C_1_ products on SACs are hardly to couple with each other for C_2+_ formation. For instance, the bulk/nano Cu catalysts show high C_2+_ selectivity while only C_1_ can be be detected on the Cu SACs. Moreover, the electronic structure of single atoms is senstive to the coordination numbers of central atoms due to the quantum size effect, where the confined electrons will lead to the energy level increase and further modify the catalytic performance.^[^ ^16^ ^]^ The fine structure of SACs are usually characterized by various technologies, such as Cs‐corrected scanning transmission electron microscopy (STEM), X‐ray absorption near‐edge structure (XANES), extended X‐ray absorption fine structure (EXAFS), etc. Beneficial from these advanced technologies, the modified localized environment of SACs can be finely studied, further shedding light on desired catalysts design. Based on the physicochemical information from those technologies, the density functional theory (DFT) can be successfully used to predict experimental performance, especially in localized electro‐magnetic field manipulating on SACs. Because of this, it is believed that various SACs, dizygotic atoms catalysts, and triple atoms catalysts have exciting performance in ECR.

In a typical ECR, CO_2_ transfers from the electrolyte to the surface of catalyst follwed by activation and reduction in the active sites on the cathode.^[^ ^17^ ^]^ An optimal catalyst for the ECR not only owns high selectivity for the target products at low overpotential but also allows a large current density and long‐term stability in practical application.[^3^, ^18^] With mature sythesis methods and exact structure, metal‐based catalysts including Au, Ag, and Cu^[^ ^19^ ^]^ are attractive for fundamental studies as well as for the actual implementation in an electrolyzer. Metal‐based catalyst can be divided into four groups according to the main ECR products,[^17^, ^20^] primarily through d‐band theory and diverse binding energy between catalyst and specific chemical intermediates. The group including Pb, In, Sn bind to *H and CO_2_ intermediates weakly and thus act as a source of electrons when cathodically polarized, So the major product is formate from ECR. In contrast, the binding energy is very strong between metals (including Ni, Fe, Pt) and *CO so that catalyst generate H_2_ as the primary product, because the generated *CO covers the active sites and hampers further reduction of replenished CO_2_. The other metals and alloys, such as Au, Ag, Zn, and their alloys,^[^ ^21^ ^]^ which have a reasonable interaction with the ECR, benificial to both absorption of *CO and desorption of CO on the catalyst, are mainly used to produce CO. Cu is listed separately outside other groups as a result of it binds *CO optimally to produce varied products from CO_2_. Cu has the ability to produce products beyond CO versus other metals. Studies show that more than 16 different species are generated by polycrystalline copper foils.^[^ ^22^ ^]^ The relationship between product and material, coupled with mechanistic understandings, enables the rational design of materials for ECR.^[1a]^ Numerous works have been done to the design of metal‐based catalyst in either its structure or composition for ECR.[^21^, ^23^] However, metal‐based catalysts for ECR still suffer from certain drawbacks.[^21^, ^24^] Active sites in metal‐based catalyst are not very clear due to their complex structure and components of surface and thus the catalyst cannot be well designed at the molecular scale.^[^ ^25^ ^]^ Futhermore, their practical applications are precluded by the relatively low energetic and cost efficiency.^[^ ^26^ ^]^

There are a few pre‐eminent reviews focusing on SACs for ECR, such as the structure and synthesis of SACs, the understanding of reaction mechanism, and the commercial application perspective.^[^ ^12^, ^25^ ^]^ In this review, we focused on the strategies of effective regulation of SACs for ECR, including regulating central atoms and regulating coordination environment. We emphasized the relationship between structure and performance of SACs, especially in the carbon‐based materials. Finally, we put forward our perspectives about the opportunities and challenges of SACs in ECR system.

## 2 Support and Structure of Active Sites

**Figure 2:**
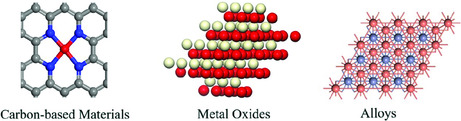
Main support of active sites of SACs.

Before the discussion on the strategies of improving the performance of ECR on SACs, it is indispensable to review support and structure of active sites in SACs, because the electronic state can be affected by the support. The electronic state variation of SACs determines the interaction between adsorption CO_2_ and active sites, thus further influces the CO_2_ coversion reaction. A rational support for ECR usually meet three conditions including good conductivity, large enough specific surface area, and sufficient stability and binding energy with single atoms.^[^ ^25^ ^]^ Recently, SACs support for ECR mainly consist of carbon materials,^[^ ^12^ ^]^ metallic oxides,^[^ ^27^ ^]^ and alloys^[^ ^28^ ^]^ (**Figure** **2**). Among these supports, the carbon materials show the highest attraction because of its lowest cost to meet industrial demand.

### 2.1 Active Sites on the Carbon‐Based Materials

The common carbon‐based supports include carbon tubes, graphene, and amorphous carbon.^[^ ^29^ ^]^ As conductors, carbon supports show high electrical conductivity to ensure high charge transport coefficient and large surface area to accelerate mass transfer,^[^ ^30^ ^]^ which are critical properties of the support for ECR applications. Furthermore, metal atoms can be stably fixed in the surface of the carbon support in isolation.^[^ ^31^ ^]^ In this case, it is widely used that heteroatoms such as N, S, P, and O and defect sites are introduced in carbon support for futher stabilizing SACs structure,^[^ ^32^ ^]^ such as M‐N_*x*_C_*y*_V_*z*_ (M = metal atoms, C = carbon atoms, V = vacancies in support). The favorable coordination environment of metal atoms in the surface not only prevents their aggregation but also changes the electronic and geometric structure of the metal active sites.^[^ ^33^ ^]^ Hence, no definitive structure for SACs exsit in different carbon surpport.^[^ ^34^ ^]^ Koshy et al.^[^ ^35^ ^]^ provided effective methods for exploring structure of Ni sites atomically dispersed in carbon materials using STEM, single atom electron energy loss spectroscopy (EELS), and time‐of‐flight secondary ion mass spectrometry (ToF‐SIMS). In brief, it is vital to construct a processable support at atomic level for catalyst structure.^[^ ^36^ ^]^

**Figure 3:**
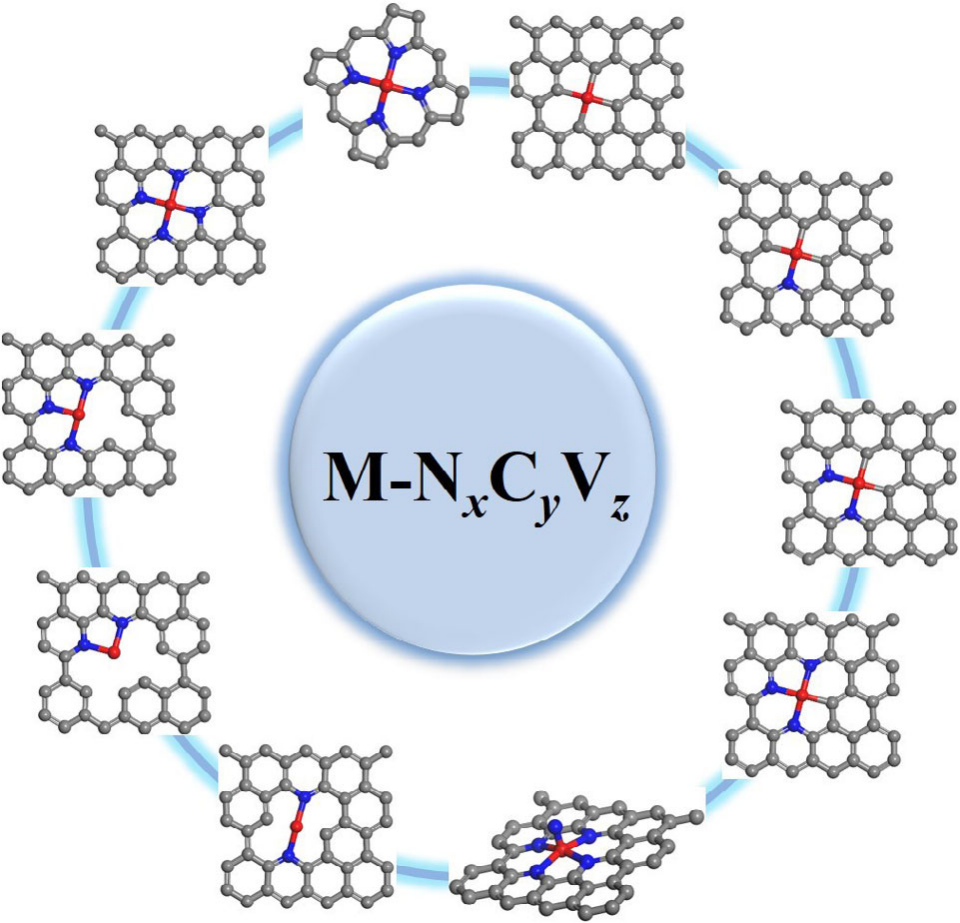
Various typical types of M‐N_*x*_C_*y*_V_*z*_ in the carbon support.

M‐N_*x*_C_*y*_V_*z*_ is the most common structure of SACs in the field of ECR as well as other electrocatalytic fields (**Figure** **3**). The synthesis of M‐N_*x*_C_*y*_V_*z*_ catalysts for ECR generally involves the bonding between metal atoms and coordination elements on precursor or carbon‐based substrates during high‐temperature pyrolysis, electrochemical deposition, and physical deposition. The various active sites, with different coordination numbers and environment in carbon‐derivatives support, are influenced by carbon and metal precursors, likewise the surounded conditions for the catalyst preparation. Three common configurations of N in N‐doped carbon materials are pyridinic‐N, pyrrolic‐N, and graphitic‐N. Single atoms mainly including transition metals both keep stable in carbon support by means of M‐N with pyridinic‐N and pyrrolic‐N. Cheng et al. prepared atomically dispersed Ni on N‐CNTs (NiSA‐N‐CNTs) by a multistep pyrolysis method.^[^ ^37^ ^]^ Each Ni atom is coordinated with four pyridinic‐N atoms. NiSA‐N‐CNTs with superhigh loading Ni atoms (20 wt%), show an extraordinary selectivity and activity for ECR to CO. Li et al. used Ni‐doped g‐C_3_N_4_ as precursor to prepare Ni‐N_4_‐C catalyst. The Ni‐N_4_ structure exhibits excellent activity for ECR with an excellent FE_co_ over 90% in a wide potential range from −0.5 to −0.9 V. The active site Ni atoms from Ni‐N_pyridinic_ exhibit a 30 h stability at 0.81 V. Yang et al.^[^ ^38^ ^]^ constructed a high‐yield, flexible, and free‐standing porous carbon membrane catalyst decorated with Ni‐N_pyridinic_ structure through electrospin for ECR. Based on 120 h stability tests in GDE cell, this catalyst exhibits a promising prospect in the industrial carbon cycling. A universal synthetic strategy is reported to allow the synthesis of various transition‐metal SACs containing M‐N_pyridinic_ structure.^[^ ^39^ ^]^ M‐N_pyridinic_ with a certain superiority for ECR to produce formate and CH_4_ with high selectivity and current density are also proved in other studies.^[^ ^15^, ^40^ ^]^

Compared with M‐N_pyridinic_, M‐N_pyrrolic_ seems to be less used but still make a difference for ECR. SACs embeded with M‐N_pyrrolic_ for ECR are prepared by three steps including polymerization, oxidation, and pyrolysis.^[^ ^41^ ^]^ The current densities of producing CO have a positive correlation with Ni loading (from 0 to 3.4 wt%) in NiPACN. Authors reported that the active sites are Ni atoms in Ni‐N_pyrrolic_. The Ni‐N_4_ structure is confirmed by spectroscopic results. This suggestes that active sites in NiPACN is identical to Ni porphyrin, a typical molecular catalyst. Thus a direct analogy can be subsistent between heterogeneous and homogeneous catalysts. A universal strategy to synthesize varieties of SACs embeded with Ni‐N_pyrrolic_ is also put forward by Zheng et al.^[^ ^42^ ^]^ Ni—N—C with isolated metal atom coordinated by four pyrrolic N atoms exhibits an extraodinary ECR performance along with long‐time stability, superior to majoriy of previously reported SACs for CO_2_‐to‐CO conversion. Other N_pyrrolic_ captured metal atoms including Fe^3+^‐N_pyrrolic_‐C^[^ ^43^ ^]^ and Cu‐SA/NPC^[^ ^44^ ^]^ are also applied for ECR. The exact structure M‐N_*x*_C_*y*_V_*z*_ makes the mechanism of ECR clearer.

### 2.2 Active Sites on the Metal Oxides

In addition to carbon materials, metal oxides with super stability and abundant ligand sites for single atoms can also serve as support for SACs.[^32^, ^45^] However, up‐to‐date, SACs containing noncopper metal atoms embedded in noncarbon sustrate are rarely reported in efficient ECR yet so far. It may be difficult for metal atoms in addition to Cu to generate catalyts that appreciate activity and selectivity for ECR. The uniqueness of Cu SACs in respect to selecting support is similar to that only Cu‐based metals and metallic compound can produce C_2_ and C_2+_.^[^ ^46^ ^]^ Wang et al. proposed a feasible method to prepare copper‐doped, mesoporous CeO_2_ nanorods for ECR to CH_4_.^[^ ^27^ ^]^ The localized evironment modulation on CeO_2_ is realized by Cu substitution, where the Cu atoms occupy the holes of Ce atoms on CeO_2_ surface, and thus promote the FE_CH4_. Theoretical calculations were conducted to verify that copper substitution in CeO_2_ (110) surface can steadily enrich up to three oxygen vacancies around each Cu site, generating very effective active sites for CO_2_ adsorption and activiation. In 0.1 m KHCO_3_, the most promising catalyst Cu‐CeO_2_–4% reduces CO_2_ to methane with a high FE_CH4_ of 58%, indicating that the motivated modulation of active sites is beneficial to promote activity and selectivity.

### 2.3 Active Sites on the Alloys

Alloys with high conductivity and electron affinity are qualified candidates for supporting single atoms.^[^ ^47^ ^]^ The high electron affinity of support can concetrate the negatively charged species, ensuring a high OH^−^ or CO_3_^2−^ cations concentration surrounding the active sites, which will highly suppress the hydrogen evolution reaction and promote the ECR activity. Single‐atom alloys are reported that the chemically active atoms are dispersed in more inert and catalytically selective host metals.^[^ ^48^ ^]^ Single‐atom alloys have been studied to catalyze a range of industrially vital reactions in electrochemical‐, photo‐, and thermal catalysis studies. The Pd_10_Te_3_ alloy nanowires are demonstrated to stably support two kinds of Cu atoms in different valence. The large current density of Cu‐APC (an atom‐pair catalyst) remained unchanged at −0.78 V after 3 h, and meanwhile, the FE_CO_ shows a descend from the initial 92% to the subsequent 80%, indicating a still relatively high CO selectivity.

## 3 Regulating Central Atoms for Improving the Performance of SACs

**Figure 4:**
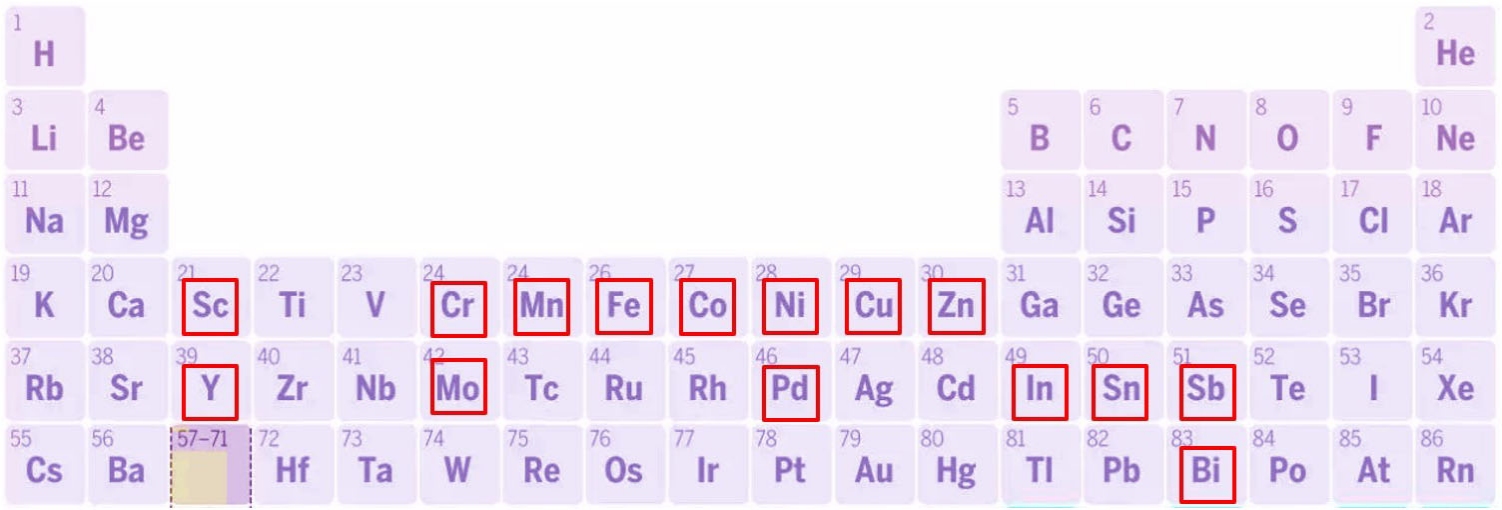
The reported central atoms in SACs for ECR.

Recently, a dozen types of atoms are used in ECR (**Table** **1**). **Figure** **4** shows the commonly used metal atoms in SACs. Regulating the central atoms is an effective method to manipulate the products of SACs during ECR, as single atoms are located in the center of the structure of SACs. Transition metal atoms are the most used in ECR, especially the Fe, Co, Ni, Cu, and Zn, because their low cost and earth‐abundant resource.^[^ ^25^ ^]^ CO is the main product of these SACs during ECR, because of the lack of C—C coupling sites on those isolated atoms. The selectivity among those SACs is determined by their intrinsic catalytic activity, such as the ability of activating CO_2_ molecules and the bonding energy between active sites and intermediate. In addition, a few part of main group elements including In, Sn, Sb, and Bi can be also used to build the SACs. Formate is the most commonly produced in those SACs. Upon considering the need of effective carbon recycling, various types of C‐based products in ECR play an important role in subsequent industrial synthesis of value‐added products. Thus, it is essential to genergate desired catalysts with processable catatlytic performance via regulating central atoms. In this part, we sumarize SACs with various central atoms and focused on the fundamental difference of central atoms in reaction mechanism.

**Table 1:** The different central atoms and structures in SACs for ECR

SACs	Structures	Main products	Loading	FE_max_@Potential vs RHE	Ref.
Fe SACs	Fe‐N_4_	CO	0.36 at%	94%@−0.58 V	[61a]
Fe SACs	Fe‐N_5_	CO	–	95%@−0.53 V	[61b]
Fe SACs	Fe‐N_4_	CO	–	80%@−0.57 V	[104]
Pd SACs	Pd‐N_4_	CO	2.95 wt%	55%@−0.50 V	[89]
Ni SACs	Ni‐N_4_	CO	2.4 wt%	97%@−0.90 V	[56]
Cu SACs	Cu‐N_2_	CO	1.45 wt%	81%@−0.50 V	[95]
Ni SACs	Ni‐N_4_	CO	–	92%@−0.68 V	[105]
Sn SACs	Sn‐N_2_C_2_	HCOOH	0.82 wt%	75%@−0.74 V versus SCE	[83]
Ni SACs	Ni‐N_2_C_2_	CO	0.9 wt%	98%@−0.8 V	[94]
Cu SACs	Cu‐N_4_	C_2_H_5_OH	0.5 wt%	55%@−1.2 V	[74]
Zn SACs	Zn‐N_4_	CO	0.10 wt%	95%@−0.43 V	[76]
Fe SACs	Fe‐N_5_	CO	–	97%@−0.35 V	[96]
Zn SACs	Zn‐N_4_	CH_4_	2.66 wt%	85%@−1.8 V	[40]
Ni SACs	Ni‐N_4_	CO	1.41 wt%	99% @ −0.81 V	[106]
Co SACs	Co−N_5_	CO	3.54 wt%	99.2%@−0.73 V	[72]
Cu SACs	Cu‐N_4_	CH_3_OH	1.4 wt%	44%@−0.90 V	[75b]
Mn SACs	Mn–N_3_	CO	0.17 wt%	98.8%@0.44 V	[88a]
Ni SACs	Ni‐N_4_	CO	1.3 wt%	96%@ −0.70 V	[38]
Ni SACs	Ni‐N_4_	CO	–	98.9%@−1.2 V	[39]
Mn SACs	Mn‐N_4_Cl	CO	0.049 wt%	97%@−0.60 V	[88b]
Cu SACs	Cu‐N_4_	CH_3_COCH_3_	0.59 wt%	36.7%@−0.36 V	[44]
Ni SACs	Ni‐N_4_	CO	2.5 wt%	97%@−0.61 V	[97]
Sb SACs	Sb–N_4_	HCOOH	2.86 wt%	94%@−0.8 V	[84]

### 3.1 Ni SACs

As Ni‐N_*x*_ endows SACs with an inherent performance for ECR, Ni SACs are nearly the most extensive and valid catalysts for converting CO_2_ to CO.[^1^, ^12^, ^25^, ^37^] The most commonly used synthesis of Ni SACs is pyrosis. The most common structure of Ni SACs consisting Ni atoms is coordinated by four pyridinic‐N atoms from the N‐doped carbon surpport.^[^ ^35^, ^49^ ^]^ Under the same conditions including preparation and measurement, Ni SACs always exhibit a more excellent ECR performance than other SACs, such as Fe, Co, Ni, and Cu.^[^ ^42^, ^50^ ^]^

Hu et al.^[^ ^51^ ^]^ synthesized M‐N‐C (Fe, Co, Ni) on N‐doped porous carbon electrocatalysts via silica‐templated pyrolysis. Results can be received from the databases of X‐ray photoelectron spectra (XPS), STEM, and EXAFS. First, the metal atoms are atomically dispersed on the carbon matrix and then M‐N_4_ is the active site in the M‐N‐C, that is, Fe‐N‐C, Co‐N‐C, and Ni‐N‐C feature identical parameters in chemical environment and substrate. Among those materials, Ni‐N‐C shows the best performance in ECR with a FE_CO_ of 93% at −0.67 V versus RHE. A consequential conclusion has been drawn that the general selectivity order of CO_2_‐to‐CO conversion is found to be Ni > Fe > Co.^[^ ^42^, ^50^ ^]^ Furthermore, Jiang et al. made a further explanation in mechanism and performance of M‐N‐C.^[50b]^ A universal synthetic method toward single‐atom metal embedded N‐doped carbon had been made based on multivariate MOFs. The M_1_‐N‐C (M = Fe, Co, Ni, and Cu) catalysts share the same distribution and structure. Ni_1_‐N‐C exhibits higher FE_CO_ up to 96.8%, far better than Fe, Co, and Cu based M_1_‐N‐C catalyst. After building the ideal models of M‐N_4_, DFT calculations can be used to well explore the mechanism. It is believed that the rate‐determining step of M_1_‐N‐C catalysts is the formation of *COOH. Co_1_‐N‐C and Cu_1_‐N‐C exhibit much higher energy barriers than Ni_1_‐N‐C and Fe_1_‐N‐C in all investigated samples. Because of this, Ni_1_‐N‐C and Fe_1_‐N‐C manifest more extraordinary activity in ECR. Although the Fe_1_‐N‐C should show higher catalytic activity/selectivity than Ni_1_‐N‐C according the energy barrier differences of *COOH formation on metal atoms, the Ni_1_‐N‐C exhibit higher FE_CO_ in all relevant experimental resutls. To dissolve this discrepancy between the theoretical and experimental results, the desorption of CO on metal sites is studied by DFT. It is reported that the CO‐Fe bonding energy is much higher than CO‐Ni, verifying the easier CO release from Ni_1_‐N‐C. Afterward, because H_2_ and CO are detected in products only, the limiting potential difference between ECR and HER (U_L_(CO_2_)‐U_L_(H_2_); U_L_= −ΔG_0/e_) can be used as the descriptor of CO selectivity, where more positive value of U_L_(CO_2_)‐U_L_(H_2_) represents a higher ECR selectivity than hydrogen evolution.^[^ ^52^ ^]^ The U_L_(CO_2_)‐U_L_(H_2_) values of Fe_1_, Co_1_, Ni_1_, and Cu_1_‐N‐C are −1.15, −1.98, −1.19, and −2.33 eV, respectively. The results from DFT calculation are highly consistent with the experimental results. Experimentally, the catalytic activity of these SACs follows the order of Ni_1_‐N‐C > Fe_1_‐N‐C > Co_1_‐N‐C > Cu_1_‐N‐C. Mou et al. reported a catalyst named as NiSA–NGA, which is derived from graphene aerogel in four different temperatures.^[^ ^53^ ^]^ Significant difference is not dected in total current density for four NiSA–NGA electrocatalysts. NiSA–NGA–900 is confirmed as Ni SACs with a high Ni loading of ≈2.6 wt%, which shows the highest FE_CO_ of 90.2% at the overpotential of 0.69 V (electrolytic potentials of −0.8 V versus RHE). Furthermore, NiSA–NGA–900 triggers the CO_2_ reduction reaction (CRR) at a very low potential of 0.29 V (electrolytic potentials of −0.4 V versus RHE). Nevertheless, the FE_CO_ of NiSA–NGA–900 starts to decrease at more negative potentials, because mass transfer at 0.8 V versus RHE limits CRR and FE_H2_ increased. Three catalysts are prepared using this method, where the NiSA–NGA shows better performance in ECR than FeSA–NGA and CoSA–NGA.

**Figure 5:**
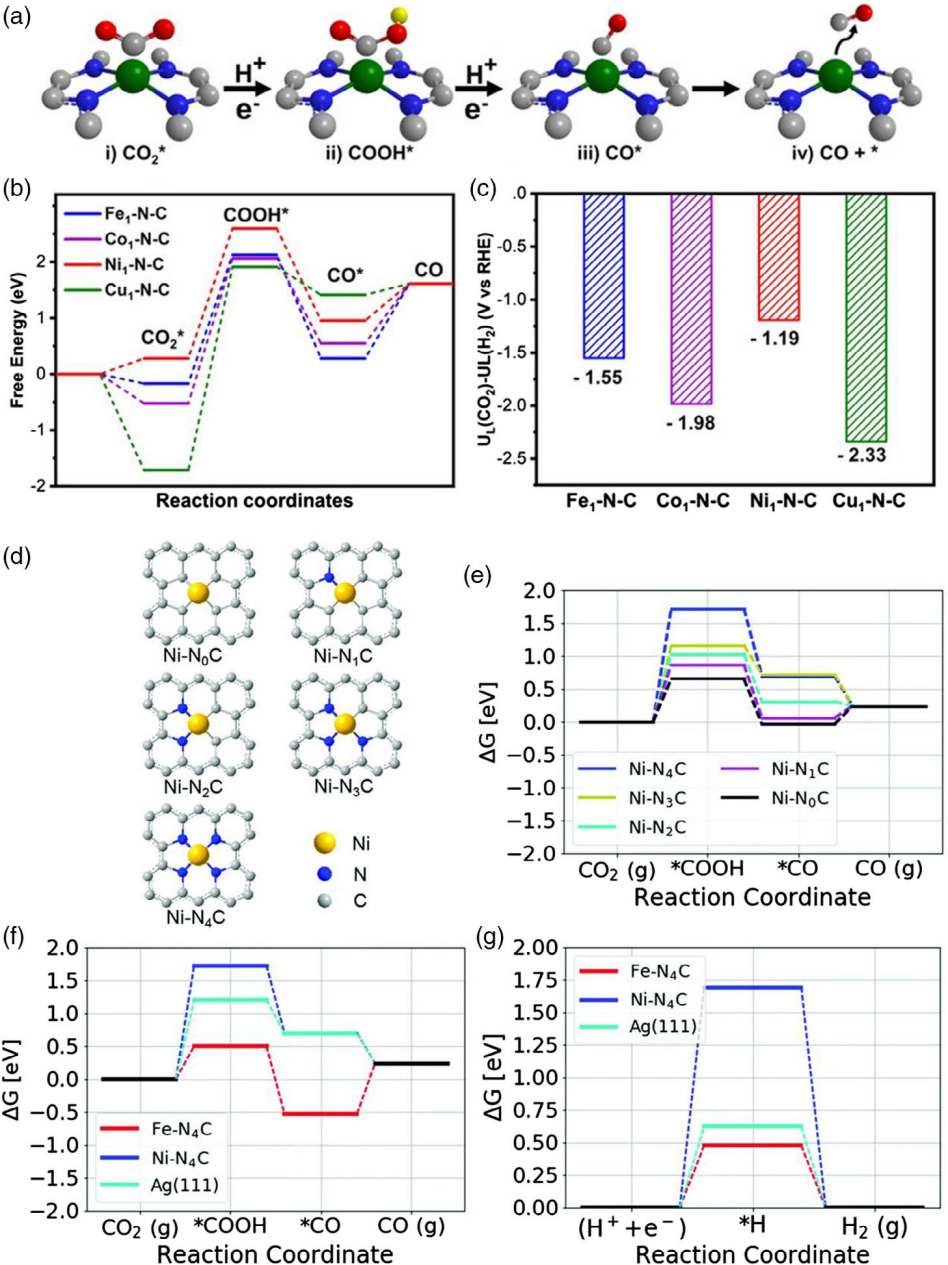
DFT calculations. a) Reaction paths, b) free energy diagrams of CO_2_ reduction to CO, and c) the values of U_L_(CO_2_)‐U_L_(H_2_) for all M_1_‐N‐C catalysts. Reproduced with permission.^[50b]^ Copyright 2020, Wiley‐VCH. Free energy diagram of CO_2_ reduction to CO on Ni–N–C and Fe‐N‐C catalysts. d) Chemical structure of the M–Nx moieties considered. e) Influence of the Ni‐coordination on the binding strength for the *COOH and *CO intermediates. f) Free energy diagram of CO_2_ reduction to CO and (d) hydrogen evolution reaction g) on Fe‐N_4_‐C (red), Ni‐N_4_‐C (blue), and Ag(111) (cyan) catalysts. Reproduced with permission.^[50c]^ Copyright 2019, The Royal Society of Chemistry.

Many SACs containing Ni‐N‐C exhibit similar rate‐determining step of forming activiated *COOH in ECR[^50^, ^54^] (**Figure** **5**). Hence researchers focus on lowering energy barriers in *COOH formation to enhance the Ni SACs performance in ECR. For example, Yang and co‐workers synthesized isolated diatomic Ni‐Fe metal–nitrogen sites for synergistic electroreduction of CO_2_ by the impregnation and reduction method.^[54a]^ Results from DFT calculations reveal that the neighboring Ni sites transform into diatomic Ni‐Fe sites after the introduction of iron atom. The free energy barrier of CO_2_(g)—*COOH of Ni/Fe‐N‐C and Ni‐N‐C were 0.47 eV, much smaller than Ni‐N‐C, so reaction barrier for the formation of *COOH is decreased by change in the atomic environment. Results from electrochemical measurements are in good agreement with the theoretical one. Ni/Fe‐N‐C exhibits a maximum FE_CO_ of 98% at 0.7 V versus RHE and achieves a current density of 7.4 mA cm^−2^, which is 1.5 times higher than that of Ni‐N‐C. In addition, many SACs containing Ni‐N‐C share similar kinetics in the formation of *COOH. Tafel slopes in NiSA/PCFM.^[^ ^38^ ^]^ SAs–Ni–N–C,^[^ ^42^ ^]^ Ni_1_‐N‐C,^[50b]^ Ni/Fe‐N‐C,^[54a]^ Ni^I^‐NCNT@Ni_9_,^[54b]^ 2D Ni–NG^[^ ^55^ ^]^ samples were 117, 115, 98, 98, 96, 110 mV/decade, respectively. This indicates that the process from CO_2_(g) to *COOH is the rate‐determining step for Ni‐N‐C during ECR.^[54a]^ It is shown that Ni‐N‐C based on carbon support is very appropriate for ECR to CO commonly from these close values, about 100~120 mV/decade. Actually, Ni porphyrin‐based covalent triazine framework (NiPor‐CTF) with atomically dispersed NiN_4_ centers also showed an efficient performance for CRR.^[^ ^56^ ^]^

### 3.2 Fe SACs

**Figure 6:**
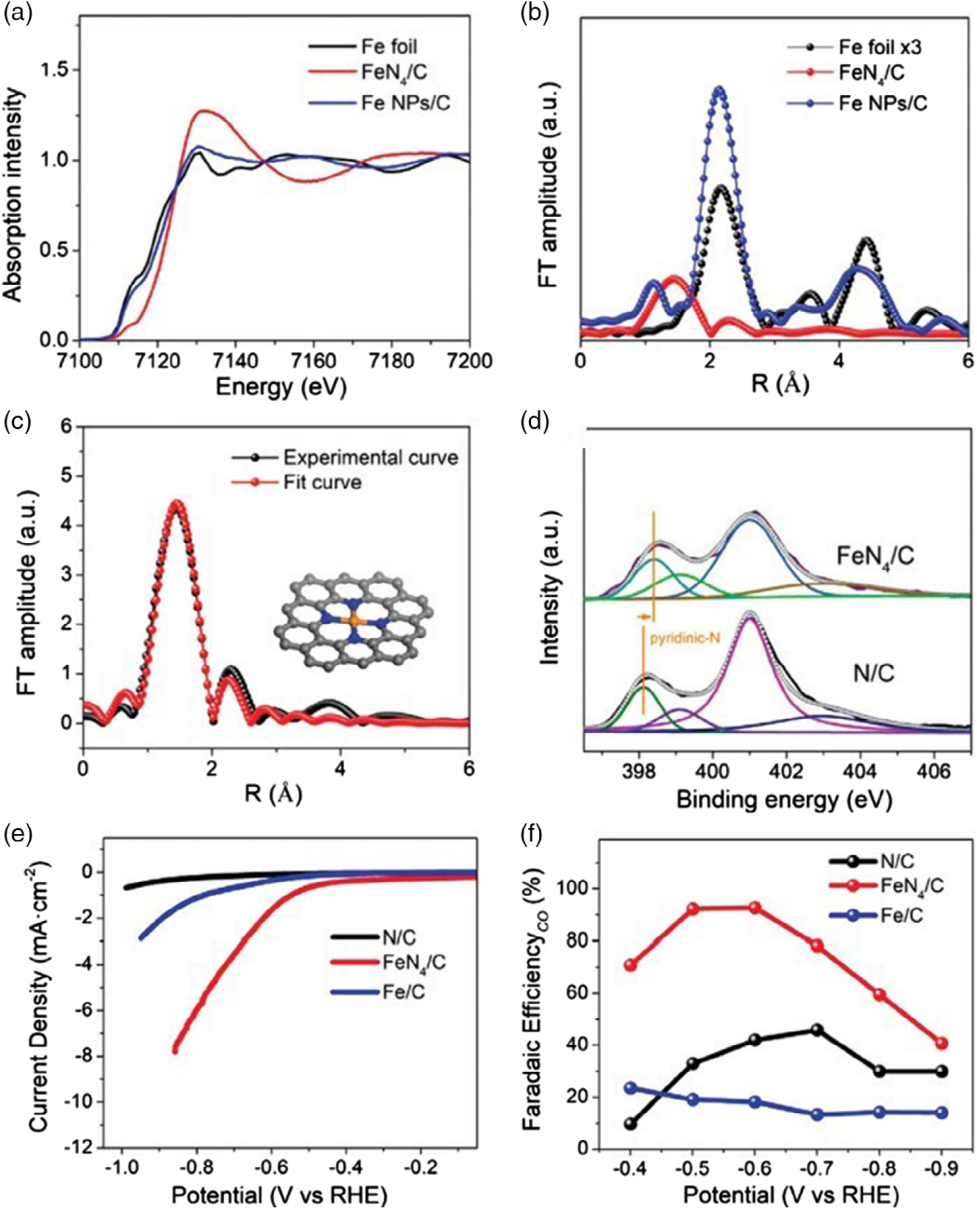
a) Fe K‐edge XANES spectra of Fe foil, FeN_4_/C, and Fe NPs/C. b) FT of the Fe K‐edge EXAFS oscillations of Fe foil, FeN_4_/C, and Fe NPs/C. c) EXAFS fitting curve for FeN_4_/C. Inset is the schematic model of FeN_4_ structure, with Fe in orange, N in blue, and C in gray. d) N 1s XPS spectra of FeN_4_/C and N/C. e) Partial current density for CO production on N/C, FeN_4_/C, and Fe/C in 0.1 m KHCO_3_. f) Nyquist plots of N/C, FeN_4_/C, and Fe/C. Reproduced with permission.^[^ ^60^ ^]^ Copyright 2020, Wiley‐VCH.

Fe SACs are usually used to fuel‐cell.^[^ ^57^ ^]^ The ECR to CO performance of Fe SACs also show desirable, ranking only second to Ni SACs counterpart in many M‐N‐C systems.^[^ ^42^, ^50^ ^]^ Fe‐N_X_ sites are responsible for the stable Fe SACs formation on N‐doped carbon support.^[^ ^58^ ^]^ Varela et al. made a preliminary exploration in Fe‐N‐C for ECR in 2015.^[^ ^59^ ^]^ Three different mono and bimetallic N‐doped carbon catalysts, known as Fe‐N‐C, Mn‐N‐C, and FeMn‐N‐C, by pyrolysizing Fe and/or Mn chloride salts, polyaniline (PANI), and Ketjenblack carbon powder were synthesized. The final metal loadings in catalysts range from 4 to 6 wt%, whereas the content of nitrogen is 6–7 wt%. Compared with the low area polycrystalline Au, M‐N‐C catalysts exhibit a 100 mV onset potential. Moreover the M‐N‐C catalysts exceed the mass activity of carbon‐supported Au catalyst at relative high CO selectivity of up to 80% at −0.5 V versus RHE. The experimental formation of methane and high FE_CO_ and CO adsorption studies provided direct mechanistic evidence. It is demonstrated that nitrogen atoms are active sites for CO production and the metal centers act as active sites for hydrocarbon formation. Later, Li et al. developed a simple adsorption method in which Fe atoms are trapped by g‐C_3_N_4_, making it an excellent template for the formation of Fe‐N_4_ sites^[^ ^60^ ^]^ (**Figure** **6**). FeN_4_/C exhibits a much higher FE_CO_ than N‐doped carbon and Fe nanoparticles, achieving a maximum FE_CO_ of 93% at −0.6 V. This work also demonstrates that the desorption of *CO is rate‐determining step, as well as other studies.^[^ ^61^ ^]^ However, actually, the exceedingly strong absorption between Fe sites and *CO rather than the free energy barrier of formation of *COOH (CO_2_(g) —*COOH), suppress continuous ECR to CO in numerous Fe SACs.[^61^, ^62^] The strong *CO binding strength results in that excessive *CO poisons the active sites and lead to FE_H2_ close to 100%.^[^ ^25^ ^]^ An optimal *CO binding strength, not too weak nor too strong, is ideal in line with the Sabatier principle.^[^ ^25^, ^63^ ^]^ Pan et al. provided a feasible strategy to improve Fe‐N‐C intrinsic reactivity.^[61a]^ Holes are constructed on graphene basal plane to support Fe−N_4_ can significantly weaken *CO binding strength so that its ECR activity is highly enhanced compared with the pore‐deficient graphene‐supported Fe−N_4_ counterpart. The binding energy is weakened from −0.92 eV (Fe‐N_4_‐pore) to −0.72 eV (Fe‐N_4_‐edge). According to the results of crystal orbital Hamilton population (COHP) and DFT calculations, they found a weakened bond strength of Fe—C between edge‐hosted Fe‐N_4_ and *CO. This is because incorporation of pore edges bring about the down‐shift of the d‐band center of Fe sites. Similar to Ni SACs.^[^ ^64^ ^]^ Fe‐N‐C usually exhibit better performance in ECR than Fe and compounds nanoparticles, but there is one exception in Fe/N‐codoped carbon electrocatalyst (FeNx/C, Fe‐N‐BCNT#BP).^[^ ^65^ ^]^ The activity of FeN_*x*_ centers is lower than that of FeC_3_, even lower than the support. In addition to CO, HCOOH and CH_3_COOH are also detected in products of ECR.^[^ ^66^ ^]^ Genovese et al. constructed an active interface between nanostructured iron (III) oxyhydroxide and nitrogen‐doped carbon. With combination of STEM, operando X‐ray spectroscopy techniques and DFT simulations, they found that Fe single atoms are formed as nitrogen‐coordinated iron (II) sites at the the edge of the graphitic layers. These Fe SACs can effectively adsorb and reduce HCO_3_^−^ species making Fe/N‐C produce. An exceptional performance of Fe/N‐C is shown in which 60.9% CH_3_COOH and 36.5% HCOOH are produced at very low potential (−0.5 V versus silver/silver chloride) during ECR.

### 3.3 Co SACs

**Figure 7:**
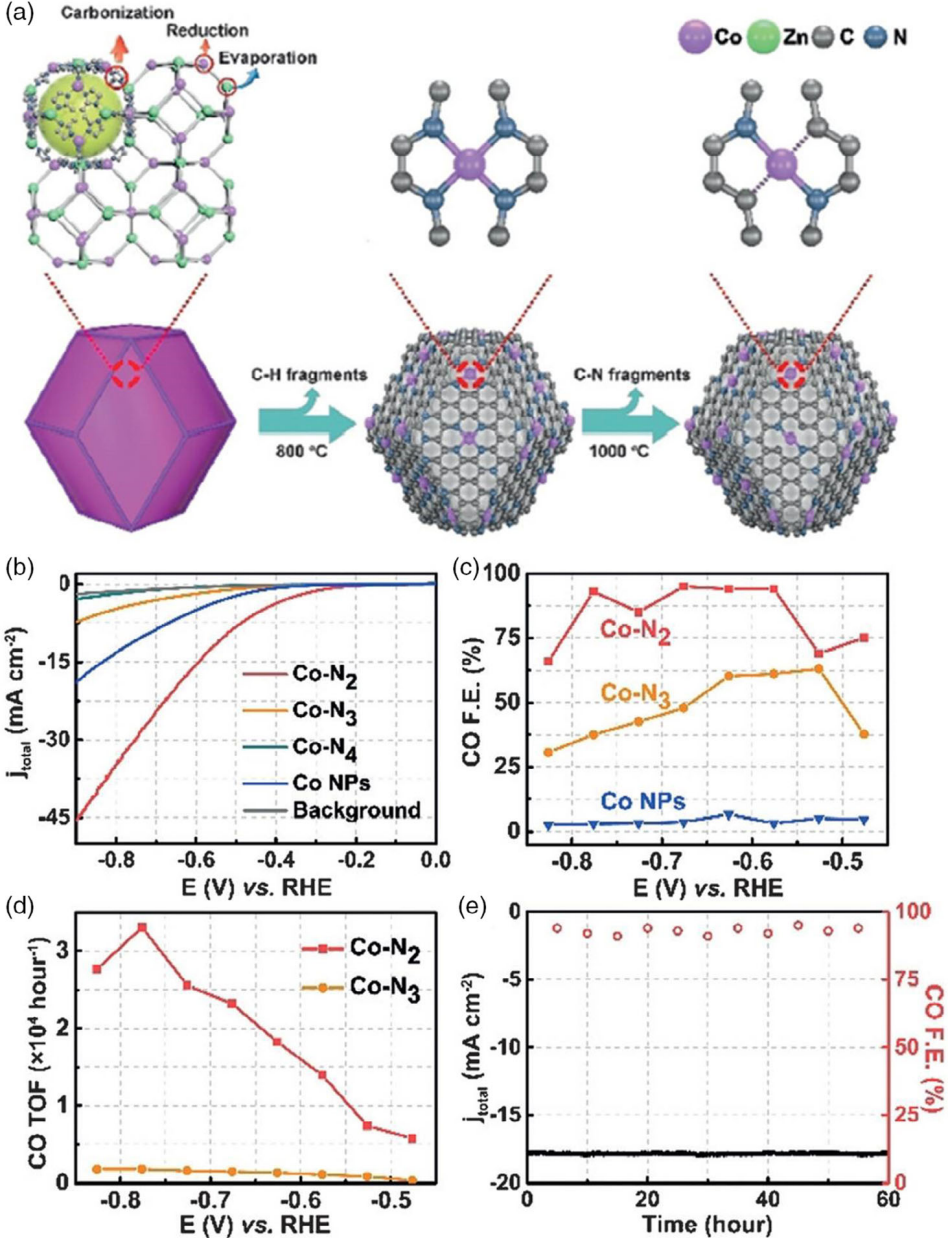
a) The formation process of Co‐N_4_ and Co‐N_2_. b) LSV of Co‐N_2_, Co‐N_3_, Co‐N_4_, and Co NPs and pure carbon paper as background in 0.5 m KHCO_3_. c) CO Faradaic efficiencies at different applied potentials and d) corresponding CO TOF for different catalysts. e) Catalytic stability test at −0.63 V for 60 h. The error bars in (b) and (c) represent the standard deviations of five independent measurements. Reproduced with permission.^[^ ^68^ ^]^ Copyright 2018, Wiley‐VCH.

Co SACs as Co‐N‐C are mainly studied for CO_2_ reduction to CO. Their performances in ECR have been proved to have poorer activity compared to that of the Ni and Fe counterparts,^[^ ^51^, ^53^, ^67^ ^]^ but higher than Co nanoparticles^[^ ^68^ ^]^ (**Figure** **7**). Hou et al. synthesized Co SACs (Co‐Tpy‐C) with well‐defined Co‐N_4_‐C, through pyrolysis of Co polypyridine complex as precursor.^[^ ^69^ ^]^ In using CO_2_‐saturated 0.5 m NaClO_4_, Co‐Tpy‐C shows a much higher current density (46.6 mA cm^−2^) than that of samples of Co doped carbon and N‐doped C. Meanwhile, with ultra low loading Co atoms (0.011 wt%), Co‐Tpy‐C exhibits high FE_CO_ (>95%) in a broad potential range from −0.7 to −1.0 V (vs RHE), and even attached the maximum FE_CO_ of 98% at −0.8 V. Co‐Tpy‐C remained stable during 24 h at −0.85 V versus RHE over 80% FE_CO_. DFT calculations demonstrated that the formation of *COOH is a rate‐determining step, which is the same as the result of Tafel slope. The energy barrier of the conversion from CO_2_ to *COOH is 0.84 eV, much smaller than that of carbon substrate (1.32 eV), indicating a good performance for ECR experimental results. Another analogous study made by Yang et al.^[^ ^70^ ^]^ is based on free‐standing carbon nanofibers (HCNFs). With the active sites Co‐N_4_ decorated, HCNFs manifest high selectivity to CO formation. Furthermore, the construction of a free‐standing, cross‐linked, and high‐yield carbon membrane (denoted as CoSA/HCNFs) with continuous porous structure, can enlarge electrochemically active surface areas (ECSA) and accelerated the reactant transportation. CoSA/HCNFs have a splendid performance with a maximum FE_CO_ (97%) at −0.6 V versus RHE and maintained high FE_CO_ (>90%) in a wide potential range (−0.4 to −0.9 V). This catalyst is also studied by DFT calculations. Similar to Co‐Tpy‐C mentioned earlier,^[^ ^69^ ^]^ the formation of *COOH is also rate‐determining step for CoSA/HCNFs. Many Co SACs based on carbon support have a close Tafel slope, about 100–150.^[^ ^69^, ^70^, ^71^ ^]^ Therefore, an important image is bulit that key point to promote performance of Co‐N_4_‐C is decreasing energy barrier of formation of *COOH. Pan et al. manipulated this energy barrier by regulating the coordination number over single Co sites from 3 to 4 and 5.^[^ ^72^ ^]^ The catalysts centers, Co single atoms, with different coordination environment are prepared at different pyrolyzed temperature. The Co−N_5_/HNPCSs catalyst exhibit the most excellent performance in three samples for ECR with FE_CO_ of 99.2% and 99.4% at −0.73 and −0.79 V versus RHE and over 90% from −0.57 to −0.88 V. The FE_CO_ of Co−N_4_/HNPCSs and Co−N_3_/HNPCSs is near to 90% from −0.57 to −0.79 V. Co−N_5_/HNPCSs with Co‐N_5_ perform better than those counterparts with Co‐N_3_ and Co‐N_4_. In the results of DFT calculations, active sites of Co‐N_5_ exhibit moderate absorption to *COOH (−0.28 eV). Therefore, it is very effective to lower energy barrier during formation of *COOH for Co−N_5_/HNPCSs, in accordance with the speculation based on aforementioned works.^[^ ^69^, ^70^ ^]^

### 3.4 Cu SACs

Cu and Cu‐based materials are some of the most attractive catalysts in ECR because their abundant products in ECR,[^19^, ^73^] and so are Cu SACs.^[^ ^44^, ^74^, ^75^ ^]^ Cu SACs could mainly generate C_1_ products. Formate can be as the main C_1_ product of ECR with using of PML catalyst (a free‐standing porphyrin‐based monoatomic layers embeded Cu‐N_4_ sites.^[^ ^74^ ^]^) PML is scalably prepared with a facile solution thermal method, which is 2D porphyrin‐based material with thickness of 2.8 Å. The activity of PML with Cu‐N_4_ sites toward formate could reach a high selectivity of 80.9% at −0.7 V versus RHE. Simultaneously, 11.5% CH_4_ could be produced at same potential from PML with Cu‐N_4_ sites. When the center Cu‐N_4_ was replaced with Au‐N_4_, it generates formate and CO as major products (40.9% and 34.4% at −0.8 V, respectively). In addtion, methanol and CO are also generated as C_1_ products from Cu SACs. Yang et al. prepared a free‐standing membrane decorated with Cu‐N_4_ sites (CuSAs/TCNFs, Cu decorated through‐hole carbon nanofibers) by electrospin.^[75b]^ CuSAs/TCNFs manifest a low onset in 0.1 m KHCO_3_ and nearly pure methanol (FE = 44%) and CO (FE = 56%) are obtained at −0.9 V versus RHE. In fact, the CO can be further reduced to what is unkown because it could be methanol or C_2_. So authors conducted DFT calculations to precisely gauge the priority of yielding methanol and the enormous difficulties of yielding C_2_ on CuSAs/TCNFs. Due to *CO desorption on Cu‐N_4_ being slightly positive (0.12 eV), indicating a endergonic step, the *CO intermediate can be further reduced to methanol. As is known, the dimerization of *CO have to rely on a relative high local concentration of *CO. As Cu single atoms mostly presented as atomically dispersed sites in CuSAs/TCNFs, the C—C coupling route of these * CO intermediates was blocked completely.

**Figure 8:**
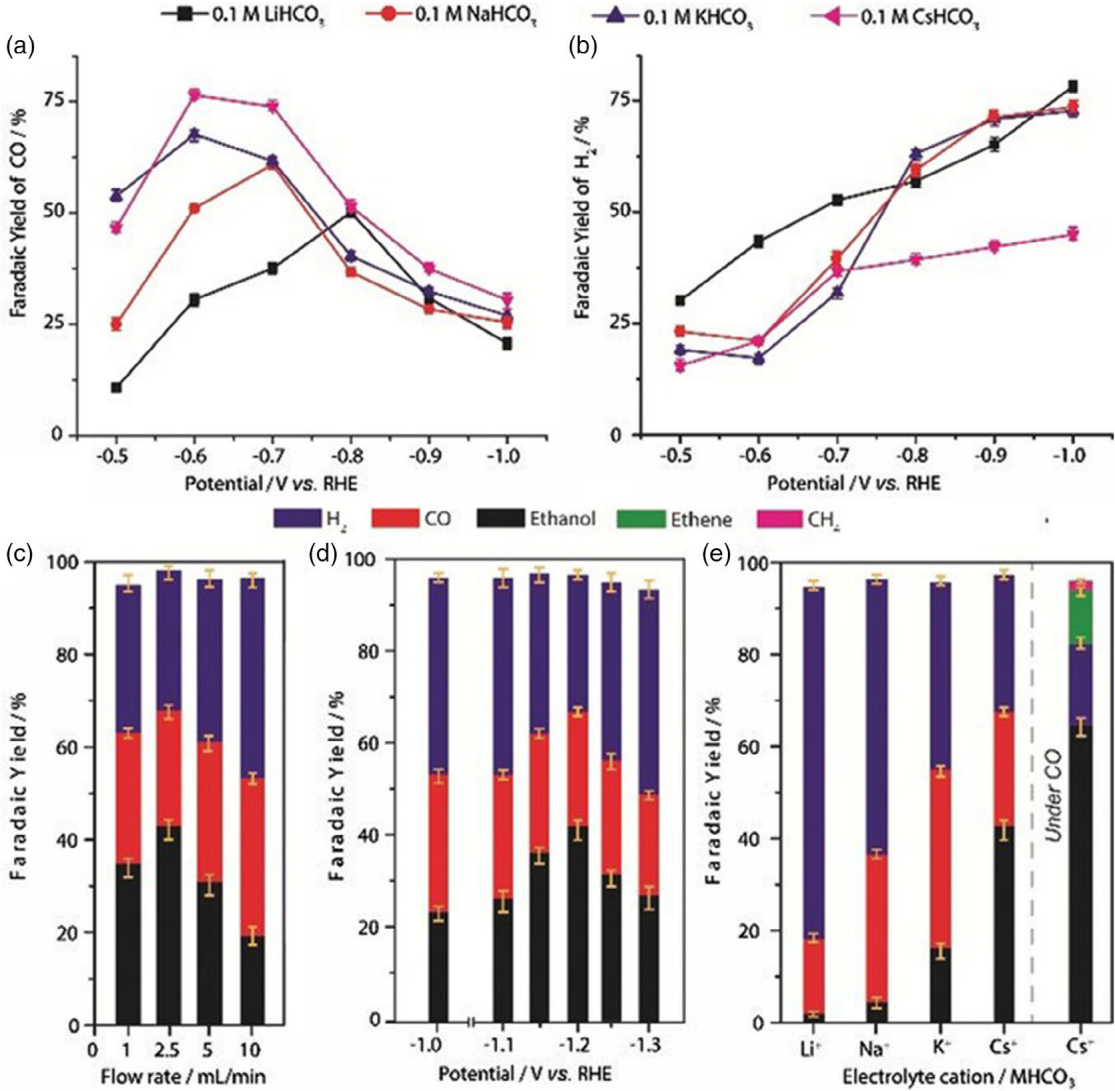
Faradaic yields of ECR on Cu_0.5_NC. Faradaic yields of a) CO and b) H_2_ under “static headspace” conditions after 10 min of CPE, in 0.1 m CO_2_‐saturated aqueous solutions of various alkali bicarbonates. FYs for ECR under “flow” conditions after 1 h of CPE: c) at −1.2 V versus RHE in a 0.1 m CsHCO_3_ aqueous solution under various flow rates of CO_2_; d) at 2.5 mL min^−1^ CO_2_ flow‐rate in 0.1 m CsHCO_3_ aqueous solution, at various applied potentials during CPE; e) at −1.2 V versus RHE and 2.5 mL min^−1^ flow rate of CO_2_ in 0.1 m aqueous solutions using various electrolyte cations. FYs of CO reduction products after 1 h CPE at −1.2 V versus RHE under 2.5 mL min^−1^ flow of CO in 0.1 m CsHCO_3_. Reproduced with permission.^[^ ^74^ ^]^ Copyright 2019, Wiley‐VCH.

In addition, Cu SACs could generate C_2_ products at high overpotential. Karapinar et al. prepared a Cu‐N_pyridine_‐C material, Cu_0.5_NC (containing 1.4 wt% Cu) for ECR to ethanol at relatively high overpotential^[^ ^74^ ^]^ (**Figure** **8**). A pyrolytic method is used to exclusively prepare Cu_0.5_NC with a Cu‐N_4_ coordination environment in a N‐doped carbon matrix. The main product from Cu_0.5_NC is CO at relatively low potential and the FE_CO_ depended on the size of the cation. The bigger the size of cation, higher the FE_CO_. When the potential increased to −1.2 V versus RHE, the main product turned into ethanol with faradic efficiency of 55% and a small amount of methane and ethylene are also observed. Intriguingly, even C_3_ (FE = 36.7%) can be obtained from Cu SACs as a major product in the study by Zhao et al.^[^ ^44^ ^]^

### 3.5 Zn SACs

**Figure 9:**
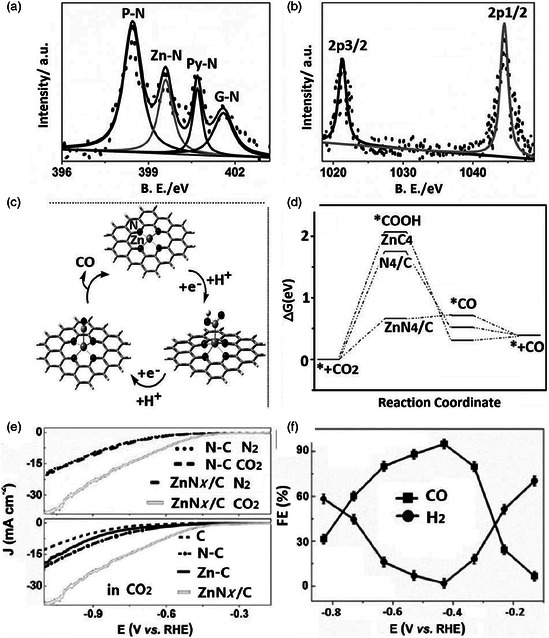
XPS spectra for N 1s in a) ZnN*x*/C and b) Zn 2p in ZnN*x*/C. c) The proposed reaction pathways for complete ECR on carbon‐supported Zn‐N_4_ active site and d) the free energy diagrams for this process on ZnN_4_/C, N_4_/C, and ZnC_4_. e) Top: pH‐corrected LSV of N‐C and ZnN*x*/C in N_2_‐saturated KH_2_PO_4_/K_2_HPO_4_ (pH 7.0) and CO_2_‐saturated (pH 7.2) 0.5 m KHCO_3_ solution; bottom: Comparison of LSV results for C, N‐C, Zn‐C, and ZnN*x*/C catalysts in CO_2_‐saturated 0.5 m KHCO_3_. f) FEs of CO and H_2_ at various applied potentials on ZnN*x*/C catalyst. Reproduced with permission.^[^ ^76^ ^]^ Copyright 2018, Wiley‐VCH.

Zn SACs are typically synthesized with zeolitic imidazolate framework (ZIF‐8) as the precursor. In general, there are two major functions of Zn single atoms for SACs in the field of ECR. 1) as a catalyst to generate CO^[^ ^76^, ^77^ ^]^ or CH_4_.^[^ ^40^, ^78^ ^]^ 2) only as an additive to optimize catalyst.^[^ ^79^ ^]^ Yang et al. successfully prepared Zn SAC (ZnN*x*/C with Zn 0.10 wt%) that contained Zn‐N_4_ sites and exhibited a remarkable performance in ECR to CO^[^ ^76^ ^]^ (**Figure** **9**). Onset overpotential of 24 mV, large turnover frequency (TOF, up to 9969 h^−1^) and high selectivity (FE_CO_ of 95% at −0.43 V versus RHE) are obtained in 0.5 m KH_2_PO_4_/K_2_HPO_4_ (pH 7.0). ZnN*x*/C also shows robust durability that the FE_CO_ and current density remain steadily at around 95% and 4.8 mA cm^−2^ during 75 h. The main active sites are confirmed to be Zn‐N_4_ by DFT calculations. The energy barrier of *COOH formation as rate‐limiting step for ECR is 0.662 eV, remarkably lower than N‐doped carbon and Zn‐C. Chen et al.^[^ ^77^ ^]^ came to similar conclusions on different substrates to the aforementioned,^[^ ^76^ ^]^ whereas Han et al.^[^ ^40^ ^]^ found that the main product is CH_4_ not CO at relatively negative potential. SA‐Zn/MNC (Zn‐N_4_ embedded in microporous N‐doped carbon) catalyzes efficient formation of CH_4_ from −1.45 to −1.9 V versus SCE. Impressively, its maximum value of FE_CH4_ is 85% at −1.8 V and constantly maintains 84% at 39.9 mA cm^−2^ after 35 h, which keeps up with performance of the Cu catalyst.^[^ ^80^ ^]^ The formation of *CHO is the rate‐limiting step for SA‐Zn/MNC in ECR to CH_4_. Zn SACs also play an important role to form more micropores and mesopores on carbon due to the evaporation of Zn at high temperature.^[^ ^81^ ^]^ Other than catalyst, Zn SACs also functioned as a spacer to prevent the aggregation of Ni and pore former in the construction of the porous structure.^[79a]^

### 3.6 Other SACs

Recently, except the aforementioned metal SACs, there are some new SACs for ECR. Sn SACs^[^ ^82^ ^]^ with the sites of Sn‐N are found to promote conversion of CO_2_ to CO with a high FE of 91% at a low overpotential of 490 mV in 0.1 m KHCO_3_. Sn SACs also play an important role in the conversion of CO_2_ to formate with the maximum FE value of ≈75% at 0.74 V versus SCE.^[^ ^83^ ^]^ Major products from Sn SACs completely differ from Sn metal. Jiang et al. demonstrated that the formation HCOO* via the first proton–electron pair addition is more favorable than *COOH formation by DFT calculations, so the major product is formate of Sb SACs which consisted of Sb–N_4_ anchored on N‐doped carbon nanosheets.^[^ ^84^ ^]^ This catalyst can serve ECR to produce formate with FE of 94.0% at −0.8 V versus RHE. Shang et al. designed In SACs containing exclusive isolated In^δ+^‐N_4_ sites on N‐doped carbon matrix derived metal–organic frameworks (MOFs).^[^ ^15^ ^]^ This catalyst showed an immensely high TOF up to 12 500 h^−1^ at −0.95 V versus RHE and the FE of formate is up to 96% with a current density of 8.87 mA cm^−2^ at low potential of −0.65 V versus RHE. Bi SCAs and metal are also disparate in the aspect of products of ECR. Exclusive Bi SCAs,^[^ ^85^ ^]^ constructed in bismuth‐based metal−organic framework (Bi‐MOF)‐derived porous carbon networks, exhibit highly efficient conversion of ECR to CO with FE_CO_ of 97% and TOF of 5535 h^−1^ at a low overpotential of 0.39 V versus RHE. The obvious advantage of Bi SCAs is the rapid formation of key intermediate *COOH with a low free energy barrier, which is demonstrated by DFT calculations. But Yang et al. found that the major product of Bi SACs is formate.^[^ ^86^ ^]^ Mn SACs usually have a high energy barrier for ECR to CO.^[^ ^87^ ^]^ However, recently, Mn SACs were also found to play a vital role in ECR to CO.^[^ ^88^ ^]^ Feng et al. prepared Mn SACs with Mn‐N_3_ site embedded in graphitic carbon nitride.^[88a]^ The ECR performance of 98.8% of FE_CO_ with a j_CO_ of 14.0 mA cm^−2^ at a low overpotential of 0.44 V is realized by Mn SACs, outperforming all reported Mn SACs before. The key factor of high efficiency of FE_CO_ was mainly that the Mn‐N_3_ site facilitated the formation of the key intermediate *COOH according to the results of in situ XPS and DFT calculations. In addition, exciting performance of ECR are also brought by Pd,^[^ ^89^ ^]^ Mo,^[^ ^90^ ^]^ rare earth element Y and Sc^[^ ^91^ ^]^ SACs. Theoretical calculations show that noble metal SACs are candidates for ECR, but experimental demonstrations are still lacking.^[^ ^92^ ^]^

## 4 Regulating the Coordination Environment

The catalytic performance of SACs is highly sensitive to their coordination environment, in which, it plays an important role in adjusting the valence state and the adsorption/desorption efficiency of active species or intermediate. The coordination environment manipulation can be realized by regulating coordination number, regulating coordination atoms and diatomic strategies. Strategies for regulating coordination environment includes regulating coordination number, regulating coordination atoms, and diatomic strategies.

### 4.1 Regulating the Coordination Number

**Figure 10:**
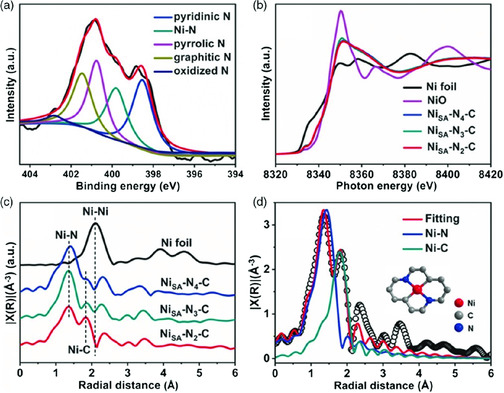
a) XPS spectrum of N 1s for NiSA‐N_2_‐C. b) Normalized Ni Kedge XANES spectra and c) FT‐EXAFS spectra of NiSA‐N*x*‐C and Ni foil. d) EXAFS fitting and optimized model for NiSA‐N_2_‐C.Reproduced with permission.^[^ ^94^ ^]^ Copyright 2020, Wiley‐VCH.

M‐N_4_ is a typical center of the metal SACs of ECR, in which the coordination number is 4. Because some metal with M‐N_4_ exhibit activities beyond expectation for ECR, it might be a favorable solution to regulate the coordination number to improve performance. Regulating the coordination number is the most discussed in the nearest reports. Sa et al.^[^ ^93^ ^]^ made a heat treatment on phthalocyanine molecules grafted on carbon nanotube (NiPc/CNT) with the structure of Ni^2+^‐N_4_ and NiPc/CNT. The fine structural characterizations show that they transform into H‐NiPc/CNT with the structure of Ni^+^–N_3_V (V: vacancy) and Ni^+^–N_3_ after calcination. A decrease in the coordination number and the oxidation state of the Ni center are found from 4 to 3 and +2 to +1, respectively. H‐NiPc/CNT with Ni^+^‐N_3_ sites exhibits ≈4.7‐times higher TOF for ECR to CO than NiPc/CNT with Ni^2+^‐N_4_. It is also demonstrated by DFT calculations that H‐NiPc/CNT has a much lower energy barrier of the formation of *COOH than NiPc/CNT. Therefore, the method of decreasing the coordination number works both in theory and experiment. Gong et al. synthesized a series of Ni SACs (named NiSA‐N*x*‐C) with different N coordination numbers. They found that the NiSA‐N_2_‐C catalyst with Ni‐N_2_ sites have much higher FE_CO_ (98%) and TOF (1622 h^−1^) than NiSA‐N_3_‐C and NiSA‐N_4_‐C^[^ ^94^ ^]^ (**Figure** **10**). DFT calculations reveal that the drecrease in N coordination number facilitates the formation of *COOH and thus accounts for a more excellent activity of ECR. Clearly, judging from the studies above, moderate decrease in coordination number of Ni‐N_4_ might improve the performance of Ni SACs. This optimization method based on decreasing the coordination number also works for Co and Cu SACs. Wang et al. designed Co SACs with different coordination numbers from 2 to 4.^[^ ^68^ ^]^ Results from experiments and calculations demonstrate that the lower a coordination number, the higher activity of Co SAC due to the same as from Ni SACs.^[^ ^93^, ^94^ ^]^ The Co SACs with two‐coordinate nitrogen atoms achieve both high selectivity and superior activity with 94% FE_CO_ formation and a current density with a current density 18.1 mA cm^−2^ at an overpotential of 0.52 V. Zheng et al. prepared two different Cu SACs, Cu–N_2_/GN (copper coordinated with nitrogen sites trapped into graphene matrix) and Cu–N_4_/GN, with different coordination number 2 and 4, respectively.^[^ ^95^ ^]^ Cu–N_2_/GN exhibits a higher ECR activity and selectivity for CO production than Cu–N_4_/GN, because Cu–N_2_ centers can not only improve the activation of CO_2_ molecule but also accelerate the electron transfer from Cu–N_2_ sites to *CO_2_, which significantly decrease energy barrier of the *COOH formation. However, increasing the coordination number works for only Fe SACs. Zhang et al. prepared the Fe SAC, containing Fe‐N_5_ sites.^[^ ^96^ ^]^ The axial pyrrolic nitrogen ligand of the Fe‐N_5_ sites can deplete the electron density of Fe 3 d orbitals and thus reduce the Fe–CO p back‐donation, which facilitates the desorption efficiency of CO. The catalyst shows FE_CO_ of 97.0% at a low overpotential of 0.35 V, outperforming all Fe‐N_4_. The same strategy is conducted to another Fe SAC.^[61b]^ Variation in the coordination number not only improves the performance of FE of main product but also in changing the type of main product. For example, the main product of Sn‐N^[^ ^82^ ^]^ in ECR is CO, while that of Sn‐N_2_‐C_2_ is formate.^[^ ^83^ ^]^

### 4.2 Regulating the Coordination Atoms

**Figure 11:**
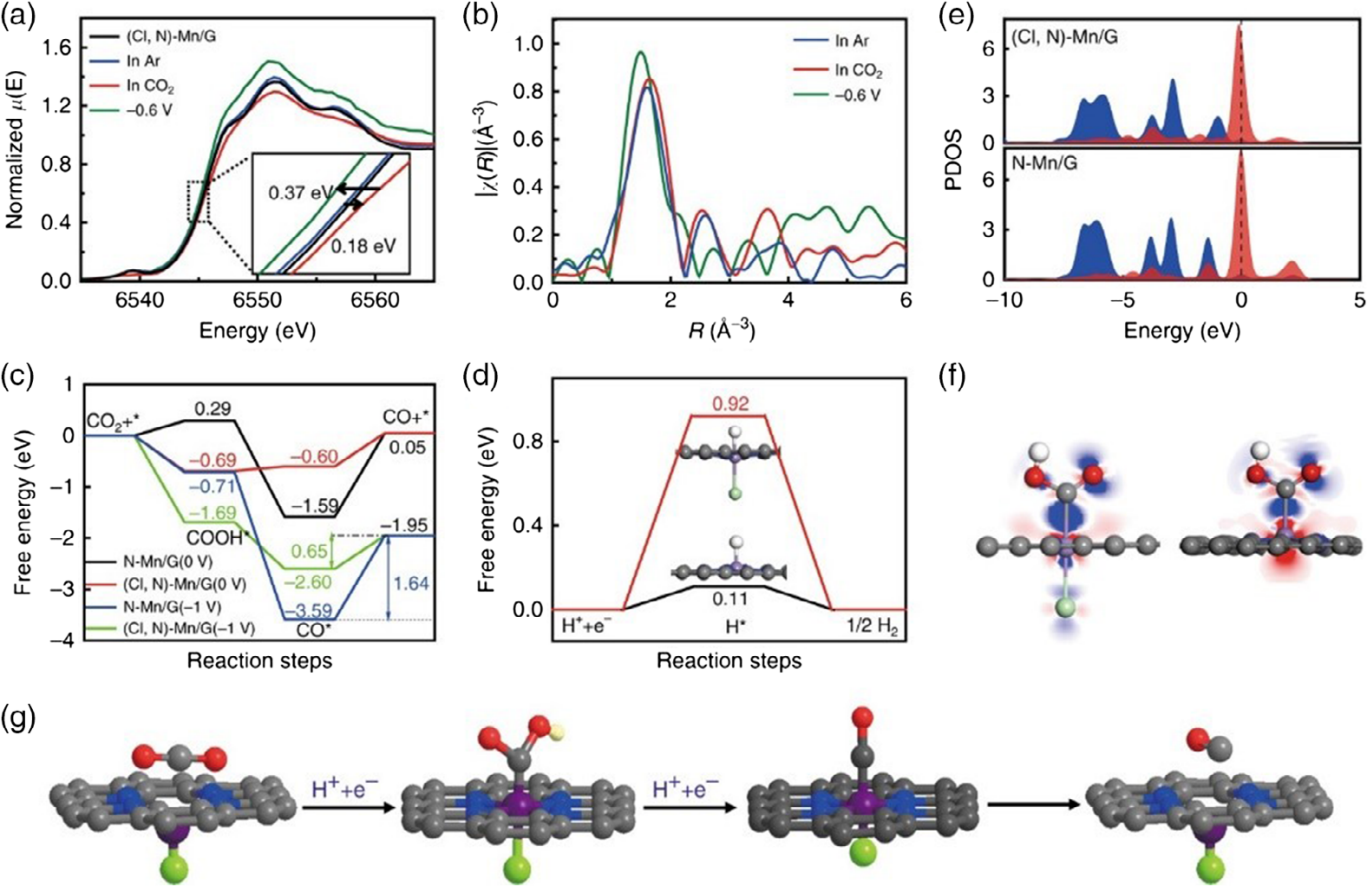
In situ XAS experiment and DFT calculation. a) Normalized XANES of (Cl, N)‐Mn/G catalyst under various conditions (inset is the magnified image). b) Fourier transform magnitudes of EXAFS spectra of (Cl, N)‐Mn/G. c) Calculated free energy of ECR. d) Calculated free energy of hydrogen adsorption. e) Projected density of states (PDOS) of the *COOH 2p state (blue‐shaded areas) and d‐projected DOS of Mn (red‐shaded areas) in the adsorption structure for (Cl, N)‐Mn/G and N‐Mn/G, respectively. f) Electron density difference for *COOH adsorbed on (Cl, N)‐Mn/G (left) and N‐Mn/G (right). The blue and red denote the electron accumulation and electron depletion, respectively. g) Structural evolution of the active site for (Cl, N)‐Mn/G in electrochemical ECR (Mn: purple, Cl: green, N: blue, O: red, H: white, and C: gray). Reproduced with permission.^[88b]^ Copyright 2019, Springer Nature.

The typical coordination atoms in SACs are nonmetallic N and C. Recently, some studies focused on the effects of different coordination atoms on the ECR performance. Zhang et al. found that when axial Cl atoms are grafted into the Mn‐N_4_ site, Mn SACs with Mn‐N_4_Cl sites exhibit an extraordinary performance with a maximum FE_CO_ of 97% and high current density of ≈10 mA cm^−2^ at a low overpotential of 0.49 V, superior to the catalyst with Mn‐N_4_ site^[88b]^ (**Figure** **11**). This modulation of the electronic structure of active sites substantially facilitates CO desorption process, which is demonstrated through in situ synchrotron X‐ray absorption spectra (XAS) and theoretical studies. In general, the variations of the coordination atoms will lead to the valence fluctuation of central metal atoms. Yang et al. developed a S‐doped Ni SACs consisting of isolated, high‐density, low‐valence Ni(i) anchored on an N‐doped graphene matrix as a robust and efficient electrocatalyst for ECR.^[^ ^97^ ^]^ Doped S facilitates the formation of the monovalent Ni(i) atomic center. The delocalization of the unpaired electron in the Ni 3d_*x*_^*2*^ *−*_*y*_^*2*^orbital and spontaneous charge are transfered from Ni(i) to the carbon 2p orbital in CO_2_ to form a CO_2_^δ−^ species, which are benificial to reduce the energy barrier for ECR. In additon, the valence of metal atoms can be influenced even by the transformation of the type of N atom in M‐N‐C. The type of N atom plays a vital role in the atomically distributed Cu anchored on N‐doped porous carbon (Cu‐SA/NPC).^[^ ^44^ ^]^ The Δ*G* value for the key step, first C—C coupling on Cu‐pyrrolic‐N_4_ and Cu‐pyridinic‐N_4_ sites, is −1.23 and 1.67 eV, respectively. An exothermic Δ*G* value on Cu‐pyrrolic‐N_4_ indicates a quite facile C—C coupling of *CO species catalyzed by the Cu‐pyrrolic‐N_4_ site. This strategy also works for Fe SACs. The Fe‐N_pyrrolic_‐C sites^[^ ^43^ ^]^ with Fe^3+^ as the active sites show more efficient performance in ECR than Fe‐N_pyridine_‐C site. Electrochemical data suggests that their superior activity derives from faster CO_2_ adsorption and weaker CO absorption than that of conventional Fe^2+^ site, which is beneficial to reduce the energy barrier of rate‐determining step of Fe‐N‐C (desorption of CO).

### 4.3 Diatomic Strategies

**Figure 12:**
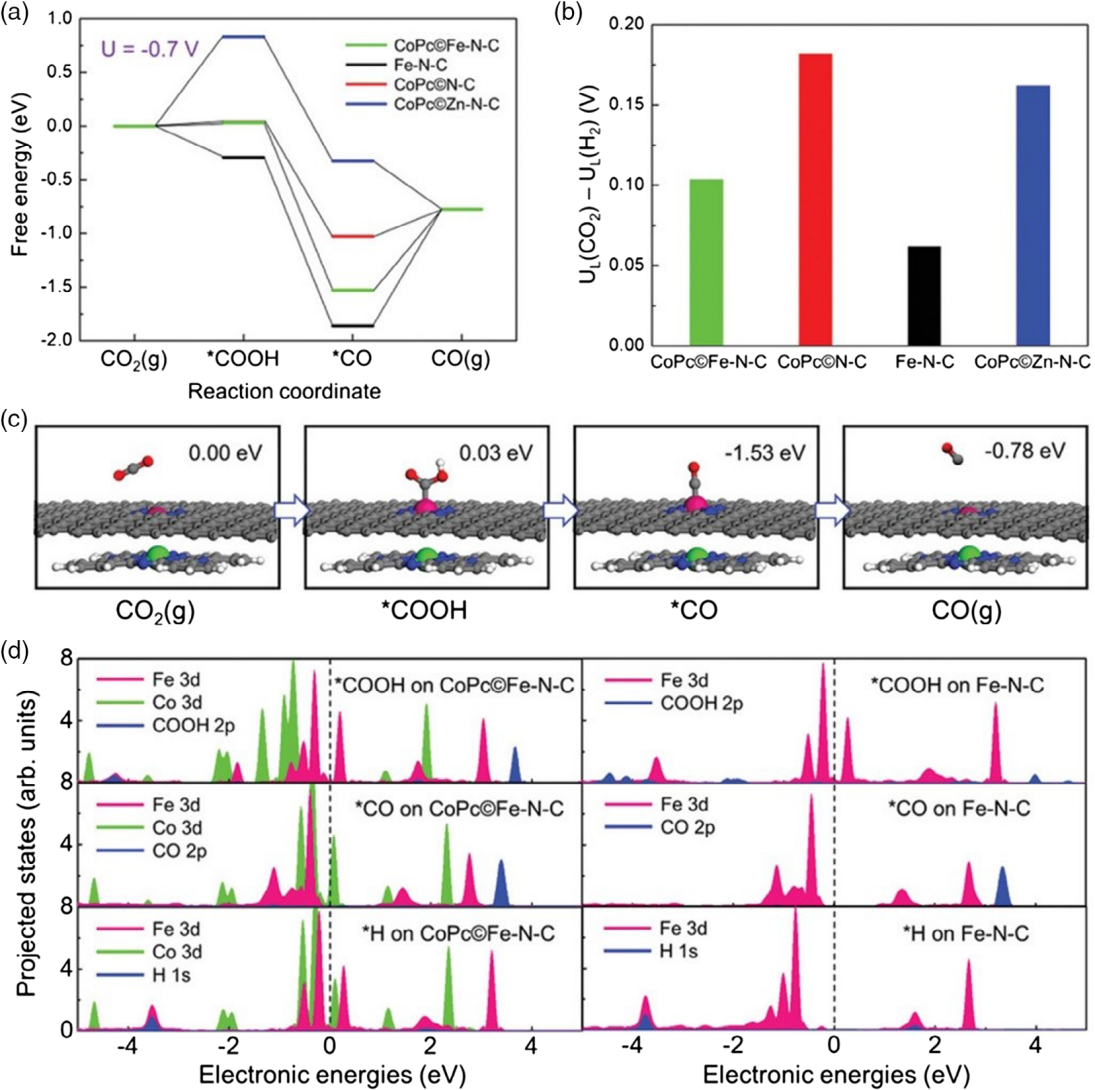
Computational results of ECR process on different catalysts. a) Free energy diagram for the ECR to CO at U = −0.7 V versus RHE on the Fe site in CoPc@Fe‐N‐C, Fe site in Fe‐N‐C, Co site in CoPc@N‐C, and Zn site in CoPc@Zn‐N‐C, respectively. b) The differences in limiting potentials for ECR (U_L_(CO_2_)) and HER (U_L_(H_2_)) on the active sites in (a). c) Schematic atomic structure of ECR process on the Fe site in CoPc@Fe‐N‐C with the free energies at U = −0.7 V versus RHE (the green line in (a)). (C: gray; N: blue; O: red; H: white; Fe: pink; Co: green). d) PDOS of adsorption structure of *COOH, *CO, and *H on CoPc©Fe‐N‐C and Fe‐N‐C, with adsorbates on Fe site: p‐density of states projected onto adsorbed *COOH and *CO, s‐density of states projected onto adsorbed *H, and d‐density of states projected onto Fe and Co atoms. Reproduced with permission.^[^ ^62^ ^]^ Copyright 2019, Wiley‐VCH.

The SACs with dizygotic atoms have risen as another new type of catalysts for ECR, benefitting from their synergistic effect on atomic level, processable localized electromagetic field, and variated free energy with surface adsorbates. Lin et al. decorated Fe SACs with cobalt phthalocyanine (CoPc) to promote the CO desorption and suppress the competitive hydrogen evolution reaction over Fe‐N sites^[^ ^62^ ^]^ (**Figure** **12**). After decoration, the maximum CO current density increases ten times after CoPc covering Fe‐N sites. This surfacial modulation also promote the stability of ECR. Lin et al. designed a CoPc and zinc–nitrogen–carbon (Zn–N–C) tandem catalyst for ECR to CH_4_.^[^ ^78^ ^]^ This tandem catalyst shows 100 times in CH_4_/CO production rate more than CoPc or Zn‐N‐C alone. The results of DFT calculations and ECR show that CO_2_ is first reduced into CO over CoPc and then CO diffuses onto Zn‐N_4_ for further conversion into CH_4_, decoupling complicated ECR pathway on single active site into a two‐step tandem reaction. Furthermore, studies also indicated that CoPc not only produced CO but also increased the availability of *H over adjacent N_pyridine_ in Zn‐N_4_, which is the key step to improve the CH_4_ production rate. Ren et al. synthesized a isolated Ni‐Fe diatomic catalyst which exhibits high FE_CO_ above 90% over a wide potential range from −0.5 to −0.9 V (98% at −0.7 V).^[^ ^54a^ ^]^ This diatomic catalyst also has a high stability up to 30 h. DFT calculations demonstrate that the *COOH formation is highly facilitated by the synergistic effect of neighboring Ni‐Fe centers on atomic level. In addition, the desorption of CO is also affected by the Ni‐Fe dizygotic atoms, benefitting from the difference between Ni and Fe electron affinity and the variation of free‐energy of Ni/Fe. Moreover, the localized physicochemical structures adapts changes once the CO_2_ uptake on Ni‐Fe dizygotic atoms during ECR, further leading to the variation of electronic landscape, atomic arrangement, and localized electromagnetic field. Exemplified by Zn‐Co,^[^ ^98^ ^]^ these characteristics in dizygotic atoms catalyst can be used to promote the ECR performance. DFT calculations reveals that a novel electronic interaction between Zn/Co enhances the absorption of *COOH intermediate on Zn sites and thus promotes CO production.

## 5 Conclusion and Perspectives

In this review, we have summarized the typical SACs on various supports for ECR and throughly discussed the relationship between catalyst structure and ECR performance. Many methods have been devoloped to manufacture the defects, external ligands, and dizygotic atoms dopant as achoring sites to generate stable SACs with high ECR. Albeit the fine structure of these types SACs are hard to be precisely defined, the unique properties of these processable anchoring sites can effectively modify the localized physicochemical structures of single metal atoms, and further affect the activity, selectivity, and stability of SACs. Because of this, we emphasized the near‐term progress in manipulating coordination environment, central atoms, and support‐metal interaction. Based on these discussions, we attemped to acquire a fundamental understanding of the desired catalytic mechanism and build the correlation between localized physicochemical structures modulation and catalytic performance. Endowed with definite structure and isolated active sites, SACs are widely studied by means of the combination of experiments and theoretical calculations. SACs, exemplified by Ni SACs, exhibit high efficiency and selectivity in ECR, which are beneficial to establish an artificial sustainable carbon recycling system and carbon neutral process. These materials also may offer reliable solutions for reducing the concentration of CO_2_.

Based on these aforementioned strategies for improving the performance of ECR, SACs can be manipulated under the guidance of experiments and theoretical calculations. Development in SACs should be attained in‐depth because of their tremendous potential in ECR. Herein, some perspectives are proposed. But challenges and opportunities still exsit. The research direction of SACs for ECR in near future can be conducted in the following four aspects.

The core factor of SACs design is to control the type and distribution of specific anchoring sites on the support. However, this approach is difficult to be realized, albeit many efforts have been made in SACs synthesis. Those unsaturated sites for stabilizing metal atoms on supports may show unexpected properties because of the large difference in geometric and electronic structures. This hampers the identification of reactive sites and the cognition of specific catalytic mechanism. For example, the N‐doped carbon‐derived materials involve various nitrogen configurations, including pyridinic‐N, pyrrolic N and edge N, where they show different electron‐affinity and electron‐donating characters. In this sense, it is urgent to develop materials with controllable localized anchoring sites and tailorable active central atoms for elucidating the fundamental correlation between the active sites and reactivity of ECR. In addition, the stability of electrocatalyst is highly vital to practical applications, whether in water splitting^[^ ^99^ ^]^ or CRR. The stability of SACs mainly depend on two aspects: 1) the chemical stability of the interation between single atoms and supports. 2) the stability of the support itself. The MXenes.^[^ ^100^ ^]^ black phosphorous,^[^ ^101^ ^]^ and MoS_2_^[^ ^102^ ^]^ can be promising alternatives for single metals atoms anchoring, because of their stable and tunable nanostructure. There are already examples of successful applications using these materials for SACs.

The catalytic performance of SACs is highly sensitive to the interaction modes between support and single atoms, which is determined by the ligands strategies between supports and atoms, atomic arrangement of surface and sublayer, and the surrounded environment during ECR. Although charge transfer between the support and the active sites has been extensively studied, the high complexity of interaction modes still impedes the acquisition of the fundamental knowledge, especially in the mechanistic and quantitative studies. In general, those surface‐sensitive XPS and scanning tunneling microscopy (STM) should be useful tools to character the efficacious electron transfer between actives sites and support. One have to mention that the support has a great impact on the ECR behaviors, even directly participates in reactions (acts as adsorption sites and/or active sites) and promote the ECR performance. In this sense, more attention should be paid to mechanistic studies of supports during characteristic reaction. In another aspect, a universal image that can describe the bond strength between active sites and support is urgently required to predict the interaction modes of metal and supports, and thus accelerates the development process of new ECR catalysts with high‐performance.

SACs are highly promising in the field of ECR, but there is very limited variety of products including CO, CH_4_, few C_2_ and C_3_^[^ ^27^, ^44^ ^]^ and it is a huge challenge to develop SACs that can reduce CO_2_ to hydrocarbon fuels with high activity and selectivity. Due to the greater value of C_2_ and C_3_, there is still much room for majority of SACs which produce only C_1_. Tranditional SACs with isolated sites hardly finish C—C process alone. Two strategies might be useful in the formation of C_2_ and C_3_: i) Diatomic strategy is a promising candidate to establish two or more sites for the formation of C—C. Based on the previous diatomic strategy in a two‐step tandem reaction for producing CH_4_, multi atomic catalyst for ECR might be in favor of the design of C_2_ and C_3_ products. ii) Tandem catalysts comprised of SACs and Cu‐based catalyst, might be another candidate to produce C_2_ and C_3_.[^46^, ^103^] The formation of C—C in Cu‐based catalyst are easier than other catalysts and SACs play a part in improving efficiencies and activities of C_2_ and C_3_.

The performance of catalysts still suffer from the structural and surficial chemistry variations during chemical reactions. These variations are triggered at high applied potential, harsh reaction condition in electrolyte, chemically active sites dissollution, etc. In this case, it is very important to probe the dynamic structure evolution of active sites (isolated atoms or support) of SACs. It is also highly desired to develop more precise techniques and optimize the measurement levels to track the structural variations together with those key intermediates and products of SACs duirng ECR. In addition, the theoretical modes are also highly needed to precisely elucidate the catalytic mechanism. Such in situ and operando spectroscopy should be used to provide a unique mechanistic and molecular level understanding of the reaction. It is shown that operando Raman or FTIR can significantly improve the understanding of the reaction mechanism by detecting the reaction intermediates. But the number of these spectroscopic studies on SACs catalysts for the ECR is limited. In summary, the development of catalysts with high ECR performance is highly limited by the unclear reconstruction of catalysts during ECR and the advanced operando charaterization techniques.

## Conflict of Interest

The authors declare no conflict of interest.

## References

[1] a) H. Liu , Y. Zhu , J. Ma , Z. Zhang , W. Hu , Adv. Funct. Mater. 2020, 30, 1910534;

[2] a) R. Francke , B. Schille , M. Roemelt , Chem. Rev. 2018, 118, 4631; 29319300 10.1021/acs.chemrev.7b00459

[3] a) D. Raciti , C. Wang , ACS Energy Lett. 2018, 3, 1545‐1556;

[4] a) Y. Wu , S. Cao , J. Hou , Z. Li , B. Zhang , P. Zhai , Y. Zhang , L. Sun , Adv. Energy Mater. 2020, 10, 2000588;

[5] a) R. Paul , L. Zhu , H. Chen , J. Qu , L. Dai , Adv. Mater. 2019, 31, 1806403; 10.1002/adma.201806403 30785214

[6] a) L. Zhang , Z.‐J. Zhao , J. Gong , Angew. Chem. Int. Ed. 2017, 56, 11326; 10.1002/anie.201612214 28168799

[7] a) S. Popović , M. Smiljanić , P. Jovanovič , J. Vavra , R. Buonsanti , N. Hodnik , Angew. Chem. Int. Ed. 2020, 132, 14844; 10.1002/anie.202000617 32187414

[8] a) L. Wang , W. Chen , D. Zhang , Y. Du , R. Amal , S. Qiao , J. Wu , Z. Yin , Chem. Soc. Rev. 2019, 48, 5310; 31588933 10.1039/c9cs00163h

[9] a) M. D. Porosoff , B. Yan , J. G. Chen , Energy Environ. Sci. 2016, 9, 62;

[10] a) S. Liu , H. Yang , X. Su , J. Ding , Q. Mao , Y. Huang , T. Zhang , B. Liu , J. Energy Chem. 2019, 36, 95;

[11] M. Ding , R. W. Flaig , H.‐L. Jiang , O. M. Yaghi , Chem. Soc. Rev. 2019, 48, 2783. 31032507 10.1039/c8cs00829a

[12] Y. Cheng , S. Yang , S. P. Jiang , S. Wang , Small Methods 2019, 3, 1800440.

[13] X. Zhou , Q. Shen , K. Yuan , W. Yang , Q. Chen , Z. Geng , J. Zhang , X. Shao , W. Chen , G. Xu , X. Yang , K. Wu , J. Am. Chem. Soc. 2018, 140, 554. 29293332 10.1021/jacs.7b10394

[14] A. S. Varela , M. Kroschel , N. D. Leonard , W. Ju , J. Steinberg , A. Bagger , J. Rossmeisl , P. Strasser , ACS Energy Lett. 2018, 3, 812.

[15] H. Shang , T. Wang , J. Pei , Z. Jiang , D. Zhou , Y. Wang , H. Li , J. Dong , Z. Zhuang , W. Chen , D. Wang , J. Zhang , Y. Li , Angew. Chem. Int. Ed. 2020, 59, 1. 10.1002/anie.202010903 32876989

[16] C. Li , W. Ni , X. Zang , H. Wang , Y. Zhou , Z. Yang , Y.‐M. Yan , Chem. Commun. 2020, 56, 6062. 10.1039/d0cc00929f 32347850

[17] a) X. Li , S. Wang , L. Li , Y. Sun , Y. Xie , J. Am. Chem. Soc. 2020, 142, 9567; 32357008 10.1021/jacs.0c02973

[18] a) Z.‐L. Wang , C. Li , Y. Yamauchi , Nano Today 2016, 11, 373;

[19] a) F. Y. Gao , S. J. Hu , X. L. Zhang , Y. R. Zheng , H. J. Wang , Z. Z. Niu , P. P. Yang , R. C. Bao , T. Ma , Z. Dang , Y. Guan , X. S. Zheng , X. Zheng , J. F. Zhu , M. R. Gao , S. H. Yu , Angew. Chem. Int. Ed. 2020, 59, 8706; 10.1002/anie.201912348 31884699

[20] a) M. B. Ross , P. De Luna , Y. Li , C.‐T. Dinh , D. Kim , P. Yang , E. H. Sargent , Nat. Catal. 2019, 2, 648;

[21] a) K. Liu , M. Ma , L. Wu , M. Valenti , D. Cardenas‐Morcoso , J. P. Hofmann , J. Bisquert , S. Gimenez , W. A. Smith , ACS Appl. Mater. Interfaces 2019, 11, 16546; 30969748 10.1021/acsami.9b01553 PMC6509640

[22] L. T. Tufa , K.‐J. Jeong , V. T. Tran , J. Lee , ACS Appl. Mater. Interfaces 2020, 12, 6598. 31922383 10.1021/acsami.9b18639

[23] a) D. Higgins , A. T. Landers , Y. Ji , S. Nitopi , C. G. Morales‐Guio , L. Wang , K. Chan , C. Hahn , T. F. Jaramillo , ACS Energy Lett. 2018, 3, 2947;

[24] a) S. Sarfraz , A. T. Garcia‐Esparza , A. Jedidi , L. Cavallo , K. Takanabe , ACS Catalysis 2016, 6, 2842;

[25] T. N. Nguyen , M. Salehi , Q. V. Le , A. Seifitokaldani , C.‐T. Dinh , ACS Catal. 2020.

[26] a) D. Ren , B. S.‐H. Ang , B. S. Yeo , ACS Catal. 2016, 6, 8239;

[27] Y. Wang , Z. Chen , P. Han , Y. Du , Z. Gu , X. Xu , G. Zheng , ACS Catal. 2018, 8, 7113.

[28] J. Jiao , R. Lin , S. Liu , W.‐C. Cheong , C. Zhang , Z. Chen , Y. Pan , J. Tang , K. Wu , S.‐F. Hung , H. M. Chen , L. Zheng , Q. Lu , X. Yang , B. Xu , H. Xiao , J. Li , D. Wang , Q. Peng , C. Chen , Y. Li , Nat. Chem. 2019, 11, 222. 30664719 10.1038/s41557-018-0201-x

[29] a) X. Han , X. Ling , D. Yu , D. Xie , L. Li , S. Peng , C. Zhong , N. Zhao , Y. Deng , W. Hu , Adv. Mater. 2019, 31, 1905622; 10.1002/adma.201905622 31617633

[30] a) Q. Wang , Y. Zhang , W. Ni , Y. Zhang , T. Sun , J. Zhang , J. Duan , Y. Gao , S. Zhang , J. Energy Chem. 2020, 50, 44;

[31] a) S. Ji , Y. Chen , X. Wang , Z. Zhang , D. Wang , Y. Li , Chem. Rev. 2020; 10.1021/acs.chemrev.9b00818 32242408

[32] a) M. Babucci , F. E. Sarac Oztuna , L. M. Debefve , A. Boubnov , S. R. Bare , B. C. Gates , U. Unal , A. Uzun , ACS Catal. 2019, 9, 9905;

[33] a) Y. Yao , Z. Huang , P. Xie , L. Wu , L. Ma , T. Li , Z. Pang , M. Jiao , Z. Liang , J. Gao , Y. He , D. J. Kline , M. R. Zachariah , C. Wang , J. Lu , T. Wu , T. Li , C. Wang , R. Shahbazian‐Yassar , L. Hu , Nat. Nanotechnol. 2019, 14, 851; 31406363 10.1038/s41565-019-0518-7

[34] a) T. Liu , W. Gao , Q. Wang , M. Dou , Z. Zhang , F. Wang , Angew. Chem. Int. Ed. 2020, 59, 20423; 10.1002/anie.202009612 32692446

[35] D. M. Koshy , A. T. Landers , D. A. Cullen , A. V. Ievlev , H. M. Meyer Iii , C. Hahn , Z. Bao , T. F. Jaramillo , Adv. Energy Mater. 2020, 10, 2001836.

[36] a) H. Huang , K. Shen , F. Chen , Y. Li , ACS Catal. 2020, 10, 6579;

[37] Y. Cheng , S. Zhao , B. Johannessen , J.‐P. Veder , M. Saunders , M. R. Rowles , M. Cheng , C. Liu , M. F. Chisholm , R. De Marco , H.‐M. Cheng , S.‐Z. Yang , S. P. Jiang , Adv. Mater. 2018, 30, 1706287. 10.1002/adma.201706287 29423964

[38] H. Yang , Q. Lin , C. Zhang , X. Yu , Z. Cheng , G. Li , Q. Hu , X. Ren , Q. Zhang , J. Liu , C. He , Nat. Commun. 2020, 11, 593. 32001699 10.1038/s41467-020-14402-0 PMC6992760

[39] H. Yang , L. Shang , Q. Zhang , R. Shi , G. I. N. Waterhouse , L. Gu , T. Zhang , Nat. Commun. 2019, 10, 4585. 31594928 10.1038/s41467-019-12510-0 PMC6783464

[40] L. Han , S. Song , M. Liu , S. Yao , Z. Liang , H. Cheng , Z. Ren , W. Liu , R. Lin , G. Qi , X. Liu , Q. Wu , J. Luo , H. L. Xin , J. Am. Chem. Soc. 2020, 142, 12563. 32536159 10.1021/jacs.9b12111

[41] D. M. Koshy , S. Chen , D. U. Lee , M. B. Stevens , A. M. Abdellah , S. M. Dull , G. Chen , D. Nordlund , A. Gallo , C. Hahn , D. C. Higgins , Z. Bao , T. F. Jaramillo , Angew. Chem. Int. Ed. 2020, 59, 4043. 10.1002/anie.201912857 31919948

[42] W. Zheng , F. Chen , Q. Zeng , Z. Li , B. Yang , L. Lei , Q. Zhang , F. He , X. Wu , Y. Hou , Nano‐Micro Lett. 2020, 12, 108. 10.1007/s40820-020-00443-z PMC7770888 34138102

[43] J. Gu , C.‐S. Hsu , L. Bai , H. M. Chen , X. Hu , Science 2019, 364, 1091. 31197014 10.1126/science.aaw7515

[44] K. Zhao , X. Nie , H. Wang , S. Chen , X. Quan , H. Yu , W. Choi , G. Zhang , B. Kim , J. G. Chen , Nat. Commun. 2020, 11, 2455. 32415075 10.1038/s41467-020-16381-8 PMC7229121

[45] a) Y. Tang , Y.‐G. Wang , J. Li , J. Phys. Chem. C 2017, 121, 11281;

[46] a) Q. Hu , Z. Han , X. Wang , G. Li , Z. Wang , X. Huang , H. Yang , X. Ren , Q. Zhang , J. Liu , C. He , Angew. Chem. Int. Ed. 2020, 59, 19054; 10.1002/anie.202009277 32686303

[47] a) J.‐C. Liu , Y.‐G. Wang , J. Li , J. Am. Chem. Soc. 2017, 139, 6190; 28406020 10.1021/jacs.7b01602

[48] a) H. Wang , Q. Luo , W. Liu , Y. Lin , Q. Guan , X. Zheng , H. Pan , J. Zhu , Z. Sun , S. Wei , J. Yang , J. Lu , Nat. Commun. 2019, 10, 4998; 31676812 10.1038/s41467-019-12993-x PMC6825208

[49] a) T. Zhang , L. Lin , Z. Li , X. He , S. Xiao , V. N. Shanov , J. Wu , ACS Appl. Energy Mater. 2020, 3, 1617;

[50] a) P. Su , K. Iwase , S. Nakanishi , K. Hashimoto , K. Kamiya , Small 2016, 12, 6083; 27634486 10.1002/smll.201602158

[51] X.‐M. Hu , H. H. Hval , E. T. Bjerglund , K. J. Dalgaard , M. R. Madsen , M.‐M. Pohl , E. Welter , P. Lamagni , K. B. Buhl , M. Bremholm , M. Beller , S. U. Pedersen , T. Skrydstrup , K. Daasbjerg , ACS Catal. 2018, 8, 6255.

[52] a) C. Chen , X. Sun , X. Yan , Y. Wu , H. Liu , Q. Zhu , B. B. A. Bediako , B. Han , Angew. Chem. Int. Ed. 2020, 59, 11123; 10.1002/anie.202004226 32239780

[53] K. Mou , Z. Chen , X. Zhang , M. Jiao , X. Zhang , X. Ge , W. Zhang , L. Liu , Small 2019, 15, 1903668. 10.1002/smll.201903668 31647616

[54] a) W. Ren , X. Tan , W. Yang , C. Jia , S. Xu , K. Wang , S. C. Smith , C. Zhao , Angew. Chem. Int. Ed. 2019, 58, 6972; 10.1002/anie.201901575 30920151

[55] K. Jiang , S. Siahrostami , T. Zheng , Y. Hu , S. Hwang , E. Stavitski , Y. Peng , J. Dynes , M. Gangisetty , D. Su , K. Attenkofer , H. Wang , Energy Environ. Sci. 2018, 11, 893.

[56] C. Lu , J. Yang , S. Wei , S. Bi , Y. Xia , M. Chen , Y. Hou , M. Qiu , C. Yuan , Y. Su , F. Zhang , H. Liang , X. Zhuang , Adv. Funct. Mater. 2019, 29, 1806884.

[57] a) Q. Zhang , J. Guan , Adv. Funct. Mater. 2020, 30, 2000768;

[58] a) Y. Chen , S. Ji , C. Chen , Q. Peng , D. Wang , Y. Li , Joule 2018, 2, 1242;

[59] A. S. Varela , N. Ranjbar Sahraie , J. Steinberg , W. Ju , H.‐S. Oh , P. Strasser , Angew. Chem. Int. Ed. 2015, 54, 10758. 10.1002/anie.201502099 26227677

[60] X. Li , S. Xi , L. Sun , S. Dou , Z. Huang , T. Su , X. Wang , Adv. Sci. 2020, 7, 2001545. 10.1002/advs.202001545 PMC7507046 32995135

[61] a) F. Pan , B. Li , E. Sarnello , Y. Fei , X. Feng , Y. Gang , X. Xiang , L. Fang , T. Li , Y. H. Hu , G. Wang , Y. Li , ACS Catal 2020, 10, 10803; 10.1021/acsnano.9b09658 32330000

[62] L. Lin , H. Li , C. Yan , H. Li , R. Si , M. Li , J. Xiao , G. Wang , X. Bao , Adv. Mater. 2019, 31, 1903470. 10.1002/adma.201903470 31441152

[63] X. Qin , S. Zhu , F. Xiao , L. Zhang , M. Shao , ACS Energy Lett. 2019, 4, 1778.

[64] X. Wang , Z.‐F. Cai , Y.‐Q. Wang , Y.‐C. Feng , H.‐J. Yan , D. Wang , L.‐J. Wan , Angew. Chem. Int. Ed. 2020, 59, 16098. 10.1002/anie.202005242 32495960

[65] E. Li , F. Yang , Z. Wu , Y. Wang , M. Ruan , P. Song , W. Xing , W. Xu , Small 2018, 14, 1702827. 10.1002/smll.201702827 29323454

[66] C. Genovese , M. E. Schuster , E. K. Gibson , D. Gianolio , V. Posligua , R. Grau‐Crespo , G. Cibin , P. P. Wells , D. Garai , V. Solokha , S. Krick Calderon , J. J. Velasco‐Velez , C. Ampelli , S. Perathoner , G. Held , G. Centi , R. Arrigo , Nat. Commun. 2018, 9, 935. 29507285 10.1038/s41467-018-03138-7 PMC5838105

[67] F. Pan , H. Zhang , K. Liu , D. Cullen , K. More , M. Wang , Z. Feng , G. Wang , G. Wu , Y. Li , ACS Catal. 2018, 8, 3116.

[68] X. Wang , Z. Chen , X. Zhao , T. Yao , W. Chen , R. You , C. Zhao , G. Wu , J. Wang , W. Huang , J. Yang , X. Hong , S. Wei , Y. Wu , Y. Li , Angew. Chem. Int. Ed. 2018, 57, 1944. 10.1002/anie.201712451 29266615

[69] P. Hou , W. Song , X. Wang , Z. Hu , P. Kang , Small 2020, 16, 2001896. 10.1002/smll.202001896 32406180

[70] H. Yang , Q. Lin , Y. Wu , G. Li , Q. Hu , X. Chai , X. Ren , Q. Zhang , J. Liu , C. He , Nano Energy 2020, 70, 104454.

[71] P. Su , K. Iwase , T. Harada , K. Kamiya , S. Nakanishi , Chem. Sci. 2018, 9, 3941. 29780526 10.1039/c8sc00604k PMC5941196

[72] Y. Pan , R. Lin , Y. Chen , S. Liu , W. Zhu , X. Cao , W. Chen , K. Wu , W.‐C. Cheong , Y. Wang , L. Zheng , J. Luo , Y. Lin , Y. Liu , C. Liu , J. Li , Q. Lu , X. Chen , D. Wang , Q. Peng , C. Chen , Y. Li , J. Am. Chem. Soc. 2018, 140, 4218. 29517907 10.1021/jacs.8b00814

[73] a) S. Sen , D. Liu , G. T. R. Palmore , ACS Catal. 2014, 4, 3091;

[74] D. Karapinar , N. T. Huan , N. Ranjbar Sahraie , J. Li , D. Wakerley , N. Touati , S. Zanna , D. Taverna , L. H. Galvão Tizei , A. Zitolo , F. Jaouen , V. Mougel , M. Fontecave , Angew. Chem. Int. Ed. 2019, 58, 15098. 10.1002/anie.201907994 31453650

[75] a) Y. Jiao , Y. Zheng , P. Chen , M. Jaroniec , S.‐Z. Qiao , J. Am. Chem. Soc. 2017, 139, 18093; 29151346 10.1021/jacs.7b10817

[76] F. Yang , P. Song , X. Liu , B. Mei , W. Xing , Z. Jiang , L. Gu , W. Xu , Angew. Chem. Int. Ed. 2018, 57, 12303. 10.1002/anie.201805871 30033610

[77] Z. Chen , K. Mou , S. Yao , L. Liu , ChemSusChem 2018, 11, 2944. 29956488 10.1002/cssc.201800925

[78] L. Lin , T. Liu , J. Xiao , H. Li , P. Wei , D. Gao , B. Nan , R. Si , G. Wang , X. Bao , Angew. Chem. Int. Ed. 2020, 59, 1. 10.1002/anie.202009191 32886835

[79] a) C. Yan , H. Li , Y. Ye , H. Wu , F. Cai , R. Si , J. Xiao , S. Miao , S. Xie , F. Yang , Y. Li , G. Wang , X. Bao , Energy Environ. Sci. 2018, 11, 1204;

[80] Y.‐L. Qiu , H.‐X. Zhong , T.‐T. Zhang , W.‐B. Xu , X.‐F. Li , H.‐M. Zhang , ACS Catal. 2017, 7, 6302.

[81] P. Yin , T. Yao , Y. Wu , L. Zheng , Y. Lin , W. Liu , H. Ju , J. Zhu , X. Hong , Z. Deng , G. Zhou , S. Wei , Y. Li , Angew. Chem. Int. Ed. 2016, 55, 10800. 10.1002/anie.201604802 27491018

[82] Y. Zhao , J. Liang , C. Wang , J. Ma , G. G. Wallace , Adv. Energy Mater. 2018, 8, 1702524.

[83] X. Zu , X. Li , W. Liu , Y. Sun , J. Xu , T. Yao , W. Yan , S. Gao , C. Wang , S. Wei , Y. Xie , Adv. Mater. 2019, 31, 1808135. 10.1002/adma.201808135 30790366

[84] Z. Jiang , T. Wang , J. Pei , H. Shang , D. Zhou , H. Li , J. Dong , Y. Wang , R. Cao , Z. Zhuang , W. Chen , D. Wang , J. Zhang , Y. Li , Energy Environ. Sci. 2020, 13, 2856.

[85] E. Zhang , T. Wang , K. Yu , J. Liu , W. Chen , A. Li , H. Rong , R. Lin , S. Ji , X. Zheng , Y. Wang , L. Zheng , C. Chen , D. Wang , J. Zhang , Y. Li , J. Am. Chem. Soc. 2019, 141, 16569. 31588748 10.1021/jacs.9b08259

[86] X. Yang , Y. Cheng , L. Qin , X. Wu , Y. Wu , T. Yan , Z. Geng , J. Zeng , ChemSusChem 2020, 10.1002/cssc.202001609. 32755063

[87] a) J. Li , P. Pršlja , T. Shinagawa , A. J. Martín Fernández , F. Krumeich , K. Artyushkova , P. Atanassov , A. Zitolo , Y. Zhou , R. García‐Muelas , N. López , J. Pérez‐Ramírez , F. Jaouen , ACS Catal. 2019, 9, 10426;

[88] a) J. Feng , H. Gao , L. Zheng , Z. Chen , S. Zeng , C. Jiang , H. Dong , L. Liu , S. Zhang , X. Zhang , Nat. Commun. 2020, 11, 4341; 32859931 10.1038/s41467-020-18143-y PMC7455739

[89] Q. He , J. H. Lee , D. Liu , Y. Liu , Z. Lin , Z. Xie , S. Hwang , S. Kattel , L. Song , J. G. Chen , Adv. Funct. Mater. 2020, 30, 2000407.

[90] P. Huang , M. Cheng , H. Zhang , M. Zuo , C. Xiao , Y. Xie , Nano Energy 2019, 61, 428.

[91] J. Liu , X. Kong , L. Zheng , X. Guo , X. Liu , J. Shui , ACS Nano 2020, 14, 1093. 31934745 10.1021/acsnano.9b08835

[92] a) S. Back , Y. Jung , ACS Energy Lett. 2017, 2, 969;

[93] Y. J. Sa , H. Jung , D. Shin , H. Y. Jeong , S. Ringe , H. Kim , Y. J. Hwang , S. H. Joo , ACS Catal. 2020, 10, 10920.

[94] Y.‐N. Gong , L. Jiao , Y. Qian , C.‐Y. Pan , L. Zheng , X. Cai , B. Liu , S.‐H. Yu , H.‐L. Jiang , Angew. Chem. Int. Ed. 2020, 59, 2705. 10.1002/anie.201914977 31821685

[95] W. Zheng , J. Yang , H. Chen , Y. Hou , Q. Wang , M. Gu , F. He , Y. Xia , Z. Xia , Z. Li , B. Yang , L. Lei , C. Yuan , Q. He , M. Qiu , X. Feng , Adv. Funct. Mater. 2020, 30, 1907658.

[96] H. Zhang , J. Li , S. Xi , Y. Du , X. Hai , J. Wang , H. Xu , G. Wu , J. Zhang , J. Lu , J. Wang , Angew. Chem. Int. Ed. 2019, 58, 14871. 10.1002/anie.201906079 31368619

[97] H. B. Yang , S.‐F. Hung , S. Liu , K. Yuan , S. Miao , L. Zhang , X. Huang , H.‐Y. Wang , W. Cai , R. Chen , J. Gao , X. Yang , W. Chen , Y. Huang , H. M. Chen , C. M. Li , T. Zhang , B. Liu , Nat. Energy 2018, 3, 140.

[98] W. Zhu , L. Zhang , S. Liu , A. Li , X. Yuan , C. Hu , G. Zhang , W. Deng , K. Zang , J. Luo , Y. Zhu , M. Gu , Z.‐J. Zhao , J. Gong , Angew. Chem. Int. Ed. 2020, 59, 12664. 10.1002/anie.201916218 32227608

[99] a) Y. Guo , J. Tang , H. Qian , Z. Wang , Y. Yamauchi , Chem. Mater. 2017, 29, 5566;

[100] a) C. Cui , R. Cheng , H. Zhang , C. Zhang , Y. Ma , C. Shi , B. Fan , H. Wang , X. Wang , Adv. Funct. Mater. 2020, 30, 2000693;

[101] a) Y. Li , L. Zhang , J. Peng , W. Zhang , K. Peng , J. Power Sources 2019, 433, 226704;

[102] a) L. Li , Z. Qin , L. Ries , S. Hong , T. Michel , J. Yang , C. Salameh , M. Bechelany , P. Miele , D. Kaplan , M. Chhowalla , D. Voiry , ACS Nano 2019, 13, 6824; 31136708 10.1021/acsnano.9b01583

[103] X. Wang , J. F. de Araújo , W. Ju , A. Bagger , H. Schmies , S. Kühl , J. Rossmeisl , P. Strasser , Nat. Nanotechnol. 2019, 14, 1063. 31591526 10.1038/s41565-019-0551-6

[104] C. Zhang , S. Yang , J. Wu , M. Liu , S. Yazdi , M. Ren , J. Sha , J. Zhong , K. Nie , A. S. Jalilov , Z. Li , H. Li , B. I. Yakobson , Q. Wu , E. Ringe , H. Xu , P. M. Ajayan , J. M. Tour , Adv. Energy Mater. 2018, 8, 1703487.

[105] W. Bi , X. Li , R. You , M. Chen , R. Yuan , W. Huang , X. Wu , W. Chu , C. Wu , Y. Xie , Adv. Mater. 2018, 30, 1706617. 10.1002/adma.201706617 29575274

[106] X. Li , W. Bi , M. Chen , Y. Sun , H. Ju , W. Yan , J. Zhu , X. Wu , W. Chu , C. Wu , Y. Xie , J. Am. Chem. Soc. 2017, 139, 14889. 28992701 10.1021/jacs.7b09074

